# Quantitative Proteomic Analysis Reveals Different Functional Subtypes among *IDH*-Wildtype Glioblastoma

**DOI:** 10.1021/acs.jproteome.5c00199

**Published:** 2025-06-16

**Authors:** Michèle Amer Salem, Jean-Louis Boulay, Marie-Françoise Ritz, Florian Samuel Halbeisen, Viviane J. Tschan, Alexander Schmidt, Katarzyna Buczak, Gregor Hutter, Severina Leu

**Affiliations:** † Brain Tumor Immuno-Therapy and Biology Laboratory, Department of Biomedicine, 30262 University Hospital of Basel, University of Basel, Hebelstrasse 20, Basel 4031, Switzerland; ‡ Surgical Outcome Research Center Basel, Department of Clinical Research, 30262 University Hospital of Basel, University of Basel, Spitalstrasse 8, Basel 4031, Switzerland; § Translational Genitourinary Cancer Research Laboratory, Department of Biomedicine, University Hospital of Basel, University of Basel, Hebelstrasse 20, Basel 4031, Switzerland; ∥ Proteomics Core Facility, Biozentrum, 27209 University of Basel, Spitalstrasse 41, Basel 4056, Switzerland; ⊥ Department of Neurosurgery, University Hospital of Basel, University of Basel, Spitalstrasse 21, Basel 4031, Switzerland; # Department of Surgery, University Hospital Basel, Spitalstrasse 21, Basel 4031, Switzerland

**Keywords:** proteomics, glioma, glioblastoma, *IDH*-wildtype, biomarkers, pathway

## Abstract

*Purpose*: Proteomics of glioma have not yet provided biomarkers and pathways that would clearly discriminate glioma subgroups. *Methods*: 82 glioma biopsies were prospectively collected and classified into six subgroups defined by methylomic classification: two low-grade glioma (LGG) and four high-grade glioma (HGG) subgroups. Proteins were extracted and processed for liquid chromatography–mass spectrometry (LC–MS). Differentially expressed proteins (DEPs) between subgroups were annotated, and functional validation was performed using inhibitor response assays in subtype-positive, patient-derived glioblastoma single-cell suspensions. *Results*: 5057 proteins were quantified for each sample. Tumor grading and *IDH* mutation status were the strongest discriminators for differential expression patterns. The glioblastoma *IDH*-wildtype subgroups showed diverse patterns of functions enriched with overexpressed DEPs: translation and cell cycle/telomere regulation in proneural glioblastoma (linked to cell proliferation), actin cytoskeleton, cell adhesion, and apoptosis regulation in classical glioblastoma (migration and invasion), and mitochondrial ATP synthesis in mesenchymal glioblastoma (metabolism). The most overexpressed proteins were correlated with survival and mRNA expression data. In vitro, inhibition of these proteins led to reduced cell viability that differed among subgroups, albeit in a small patient-derived exploratory cohort. *Conclusion*: This mainly descriptive study on proteomics in glioma provides insights into subgroup metabolism and potential biomarkers for further experimental testing.

## Graphical abstract

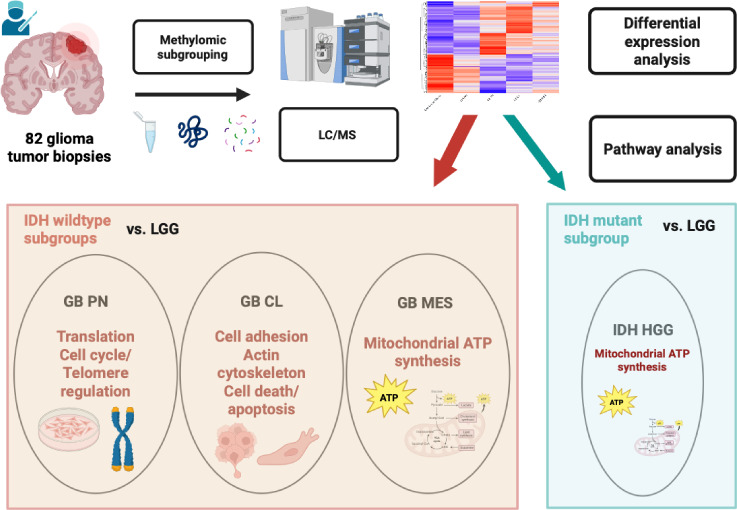

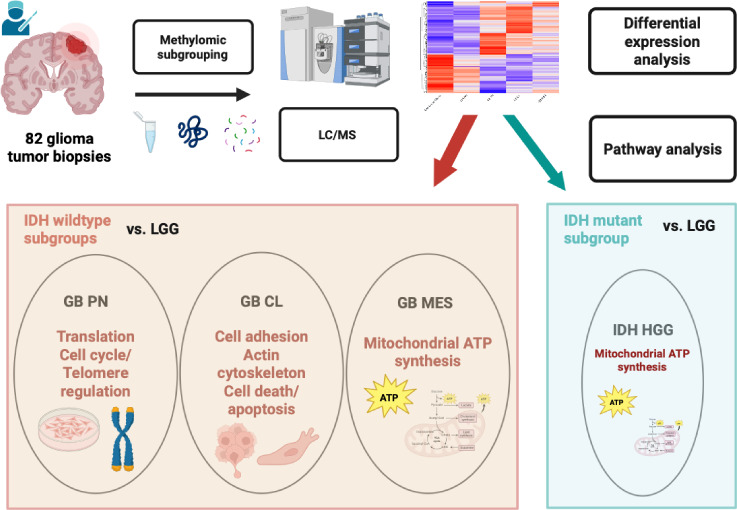

## Introduction

Gliomas are the most common primary intracranial tumors, with prognosis varying between 15 months in glioblastoma (GB), *IDH*-wildtype, and up to several decades in diffuse low-grade glioma (LGG). Cure is not possible with current therapeutic options.^1,2^ Molecular genetic approaches have led to the identification of biomarkers, such as *isocitrate dehydrogenase* (*IDH*) *1* and 2 mutations, associated with chromosome arms 1p and 19q codeletion, or *tumor protein 53* (*TP53*) mutations. These markers are being used in the World Health Organization (WHO) integrated classification of central nervous system (CNS) tumors, together with histopathological features, since 2016.^3^ This allowed the refinement of diagnosis and management of diffuse gliomas.

Earlier glioma proteomic studies have led to the identification of three molecular subgroups among GB, *IDH*-wildtype, in agreement with genomic and transcriptomic studies: proneural (GB PN), classical (GB CL), and mesenchymal GB (GB MES).^4–6^ DNA methylation profiling confirmed the subclassifications of *IDH-*wildtype as well as *IDH-*mutant gliomas into six major subgroups. The *IDH*-wildtype gliomas are GB that commonly carry chromosome 7 gain together with chromosome 10 loss.^7^ These have been further subclassified into three main subgroups: (1) the subgroup of receptor tyrosine kinase (RTK) I or proneural, (2) the subgroup of RTK II or classical, and (3) the mesenchymal subgroup.^8^ Gliomas carrying *IDH* mutations can be subdivided into (4) those with additional 1*p*/19q codeletion or low-grade oligodendroglioma (oligodendroglioma, *IDH* mutant and 1*p*/19q-codeleted), (5) those without 1*p*/19q codeletion, i.e., low-grade astrocytoma (astrocytoma, *IDH* mutant), and (6) high-grade astrocytoma, *IDH* mutant.^8^ Altogether, methylomic analysis leads to a revision of the diagnosis of 12% of all central nervous system tumors.^8^

The understanding of GB, *IDH*-wildtype subgroups has been refined by Neftel et al. in 2019.^9^ They described four different GB cellular states—mesenchymal-like (MES-like), astrocyte-like (AC-like), oligodendrocyte-progenitor-like (OPC-like), and neural progenitor-like (NPC-like) states—that coexist within one GB, *IDH*-wildtype in certain combinations and proportions, and drive GB, *IDH*-wildtype heterogeneity. Genetics and the microenvironment seem to influence the frequency of cells in each state, and in vivo single-cell lineage tracing supports the plasticity between these four states. The GB, *IDH-*wildtype subtypes reflect the highest-frequency states of each tumor and their associated genotypes: while the GB-CL and GB-MES subtypes correspond to tumors enriched for the AC-like and MES-like states, respectively, the GB-PN subtype corresponds to the combination of two distinct cellular states, OPC-like and NPC-like.^9^ However, it is important to note that the GB, *IDH*-wildtype states or subtypes are not part of the current WHO classification and do not currently possess any clinical relevance implying treatment decisions. Despite the progress made in glioma classification, no single specific biomarker that clearly distinguishes HGG subgroups from one another has been found yet. One of the reasons that could be invoked is that most studies have been detecting nucleic acid abnormalities (i.e., chromosomal aneuploidies, translocations, mutations, epigenetic dysregulation, or inappropriate RNA expression), which are predictors of molecular events taking place in tumor cells. Protein analysis quantifies effective protein levels, which are more likely to reflect the underlying oncogenic mechanisms.

Quantitative proteomic analysis aims at the identification of the protein spectrum present in each tissue sample and can therefore provide valuable insights into potentially active proteins and pathways. Many aspects of the biochemical relevance of the glioma proteome remain to be investigated and further potentially integrated into the routine diagnostic workup. To this end, we performed a large-scale quantitative proteomic analysis across cohorts of the six major subgroups of diffuse glioma from patients operated in our institution with the aim to identify specific proteins and pathways characterizing each glioma subgroup.

## Experimental Section

### Patient Identification

We screened all patients undergoing surgery for diffuse glioma between July 2017 and February 2020 in the Department of Neurosurgery at the University Hospital Basel for eligibility for this study. Patients were included in the study after histological confirmation of the presumed diagnosis and if the molecular classifier revealed a clear affiliation to one of the six glioma subgroups. All patients gave written informed consent, and ethical approval was obtained from the Ethikkommission Nordwest- und Zentralschweiz (EKNZ, Basel, Switzerland; reference: EKNZ 02019-02358).

### Tumor Tissue Collection

Tumor tissue samples of patients undergoing surgery for diffuse glioma at our institution were directly collected during surgery. The tissue was snap-frozen with liquid nitrogen and stored unembedded at −80 °C until further use.

Since the purpose of our study was a relative comparison of the proteome inventory of individual glioma subgroups against each other, non-neoplastic control tissue samples were not included.

### Data Collection

Demographic and clinical data, such as age, sex assigned at birth, tumor location, clinical presentation, surgery details, adjuvant treatments, and survival data, were extracted from our glioma tumor database or retrospectively from patient records at the University Hospital Basel. Histologic and basic molecular data were obtained from the neuropathology report.

### Tumor Subclassification

Tumors were further subclassified using genome-wide DNA methylation profile data^8^ produced at Deutsches Krebsforschungszentrum (DKFZ) as follows: “GB, *IDH*-wildtype, subtype RTK I (proneural = GB PN)”; “GB, *IDH*-wildtype, subtype RTK II (classical = GB CL)”; “GB, *IDH*-wildtype, subtype mesenchymal = GB MES”; “High-grade astrocytoma, *IDH* mutant = *IDH* HGG”; “Astrocytoma, *IDH* mutant = *IDH* LGG”; and “Oligodendroglioma, *IDH* mutant and 1*p*/19q-codeleted = *IDH* CODEL”. As the main aim of this study was the identification of protein markers specific for individual HGG subgroups, the samples from the two LGG subgroups (*IDH* LGG and *IDH* CODEL) were pooled into one single group (= LGG) used as comparator.

### Tissue Quality Control

The quality of the tumor tissue was controlled histologically by cutting several 10 μM sections from each biopsy sample with a cryostat. Sections were stained with hematoxylin and examined under a light microscope to ensure enough vital tumor cells and to exclude samples with necrosis and bleeding. Tumor biopsies from 86 patients could be used for further proteomic analysis.

### Protein Extraction and Digestion

After quality control, a 20 mg piece of tissue was cut from the biopsy sample. The piece was then transferred into a microtube and ground with a pellet pestle (Fisher Scientific, Reinach, Switzerland). The tissue was lysed in 8 M urea, 0.1 M ammonium bicarbonate, and phosphatase inhibitors (Sigma P5726 and P0044) by sonication (Bioruptor, 10 cycles, 30 s on/off, Diagenode, Belgium), and proteins were digested as described previously.^10^ Shortly, proteins were reduced with 5 mM tris­(2-carboxyethyl)­phosphine for 60 min at 37 °C and alkylated with 10 mM chloroacetamide for 30 min at 37 °C. After diluting the samples with 100 mM ammonium bicarbonate buffer to a final urea concentration of 1.6 M, proteins were digested by incubation with sequencing-grade modified trypsin (1/50, w/w; Promega, Madison, Wisconsin) for 12 h at 37 °C. After acidification using 5% trifluoroacetic acid (TFA), peptides were desalted using C18 reverse-phase spin columns (Macrospin, Harvard Apparatus) according to the manufacturer’s instructions, dried under vacuum, and stored at −20 °C until further use.

### Tandem Mass Tag (TMT) Proteomics

Sample aliquots comprising 10 μg of peptides were labeled with isobaric tandem mass tags (TMTpro 16-plex, Thermo Fisher Scientific). To enable normalization across multiple TMT sets, a representative sample pool was prepared and labeled in each TMT set (for details, see the experimental design table, Table S1). Peptides were resuspended in 10 μL of labeling buffer (2 M urea, 0.2 M HEPES, pH 8.3) by sonication, and 2.5 μL of each TMT reagent was added to the individual peptide samples, followed by a 1 h incubation at 25 °C with shaking at 500 rpm. To quench the labeling reaction, 2 μL of aqueous 0.75 M hydroxylamine solution was added, and samples were incubated for another 5 min at 25 °C with shaking at 500 rpm, followed by pooling of all samples. The pH of the sample pool was increased to 12 by adding 1 M phosphate buffer (pH 12) and incubated for 20 min at 25 °C with shaking at 500 rpm to remove TMT labels linked to peptide hydroxyl groups. Subsequently, the reaction was stopped by adding 2 M hydrochloric acid until a pH <2 was reached. Finally, peptide samples were further acidified using 5% TFA, desalted using C18 reverse-phase spin columns (Macrospin, Harvard Apparatus) according to the manufacturer’s instructions, and dried under vacuum.

### High Performance Liquid Chromatography (HPLC) Fractionation

TMT-labeled peptides were fractionated by high-pH reversed-phase separation using an XBridge Peptide Ethylene Bridged Hybrid (BEH) C18 column (3.5 μM, 130 Å, 1 mm × 150 mm, Waters) on an Agilent 1260 Infinity HPLC system. Peptides were loaded onto the column in buffer A (20 mM ammonium formate in water, pH 10) and eluted using a two-step linear gradient from 2% to 10% in 5 min and then to 50% buffer B (20 mM ammonium formate in 90% acetonitrile, pH 10) over 55 min at a flow rate of 42 μL/min. Elution of peptides was monitored with an ultraviolet (UV) detector (215 nm, 254 nm), and a total of 36 fractions were collected, pooled into 12 fractions using a postconcatenation strategy as previously described^11^ and dried under vacuum.

### Liquid Chromatography (LC)–MS/MS Analysis

Dried peptides were resuspended in 0.1% aqueous formic acid and subjected to LC–MS/MS analysis using a Q Exactive HF Mass Spectrometer fitted with an EASY-nLC 1000 (both Thermo Fisher Scientific) and a custom-made column heater set to 60 °C. Peptides were resolved using an RP-HPLC column (75 μm × 30 cm) packed in-house with C18 resin (ReproSil-Pur C18-AQ, 1.9 μm resin; Dr. Maisch GmbH) at a flow rate of 0.2 μL min^–1^. The following gradient was used for peptide separation: from 5% B to 15% B over 10 min, to 30% B over 60 min, to 45% B over 20 min, to 95% B over 2 min, followed by 18 min at 95% B. Buffer A was 0.1% formic acid in water, and buffer B was 80% acetonitrile and 0.1% formic acid in water.

The mass spectrometer was operated in DDA mode, with a total cycle time of approximately 1 s. Each MS1 scan was followed by high-collision dissociation of the 10 most abundant precursor ions, with dynamic exclusion set to 30 s. For MS1, 3e6 ions were accumulated in the Orbitrap over a maximum time of 100 ms and scanned at a resolution of 120,000 fwhm (at 200 *m*/*z*). MS2 scans were acquired at a target setting of 1e5 ions, a maximum accumulation time of 100 ms, and a resolution of 30,000 fwhm (at 200 *m*/*z*). Singly charged ions and ions with an unassigned charge state were excluded from triggering MS2 events. The normalized collision energy was set to 30%, the mass isolation window was set to 1.1 *m*/*z*, and one microscan was acquired for each spectrum.

The acquired raw files were analyzed using the SpectroMine software (Biognosys AG, Schlieren, Switzerland). Spectra were searched against a human database consisting of 20,742 protein sequences (downloaded from UniProt on 20190307 ^12^) and 392 commonly observed contaminants. The search criteria were set as follows: full tryptic specificity was required; two missed cleavages were allowed; carbamidomethylation (C) and TMT16plex (K, N-term) were set as fixed modifications; oxidation (M) and acetyl (protein N-term) were set as variable modifications. The peptide spectrum match (PSM) false discovery rate cutoff was set to 0.01. Raw reporter ion intensities of protein group-specific PSMs were exported and used for quantification.

For each TMTpro 16-plex experiment, raw PSM intensities were summed within a protein group and normalized using quantiles method.^13^ The normalization between multiple TMT experiments was performed as described here.^14^ In brief, for each protein, the geometric means of the reference channel intensities were calculated. For each TMTpro 16-plex, the protein-specific scaling was then calculated as the ratio of the geometric mean to the reference channel intensity. Protein intensities were then multiplied by the calculated scaling factors.

## Quantification and Statistical Analysis

### Statistical Analysis

We performed descriptive data analyses to summarize baseline patient characteristics. Qualitative data were summarized with the number of observations and percentages. Quantitative data were reported as the median and range. To assess the differences between tumor subgroups, Fisher’s exact tests for categorical data and Kruskal–Wallis rank sum tests for quantitative data were used. All statistical analyses were performed using R.^15^

### Identification of Differentially Expressed Proteins (DEPs)

For the analysis of the proteomics data, we included only protein samples where we had complete data. We transformed the absolute protein abundances into log_2_ values and fitted protein-wise linear models to the expression data using the Limma R library.^16^ We performed differential expression analysis to identify DEPs between subgroups and between diffuse LGG and *IDH* HGG, GB PN, GB CL, and GB MES subgroups.

Age, sex, treatments prior to surgery where the tumor sample was taken (chemotherapy, radiotherapy, steroids), and epilepsy were included as confounding factors in the model. The main aim of the study was the identification of subgroup-specific biomarkers. Gliomas are known to be contaminated with non-neoplastic cells.^17^ As a surrogate for the estimation of tumor cell content per sample, we incorporated markers associated with non-neoplastic cells (receptor-type tyrosine-protein phosphatase C (PTPRC, CD45) and HLA class II histocompatibility antigen gamma chain (HG2A) as markers for immune cells,^18^ and SEPT3 as a marker for neurons^19^ into our multivariable model to adjust for their presence. The DEPs significantly associated with these markers were excluded from the analysis. This correction aimed to align samples by neoplastic cell content and improve the comparability of protein quantification.

We used the Benjamini–Hochberg’s method to control for the false discovery rate in multiple testing. The threshold of statistical significance was set at adjusted *p* < 0.05. We then used heatmaps (package *ComplexHeatmap*),^20,21^ barplots and volcano plots (package *ggplot2*),^22^ and Venn diagrams (package *ggVennDiagram*,^23^ all in R^15^) to visualize the DEPs between our contrast groups. The heatmap displays the expression levels of each DEP, showing the variation in protein expression between the groups. Barplots and volcano plots are used to illustrate the number of over- and underexpressed DEPs. Venn diagrams show the unique and shared DEPs between the contrast groups, showing the overlap in protein expression.

### Dimensionality Reduction: Principal Component Analysis (PCA) and Uniform Manifold Approximation and Projection (UMAP)

We used PCA and UMAP to our data set of DEPs to both simplify its complexity and visualize the underlying patterns among the DEPs across various glioma subgroup contrasts. PCA effectively captured the main variations among DEPs, while UMAP provided nuanced mapping of protein expression patterns. These analyses were performed using the FactoMineR^24^ and umap^25^ packages in R, respectively.^15^

### Gene Ontology (GO) Enrichment, Kyoto Encyclopedia of Genes and Genomes (KEGG) Pathway Analysis, and Protein–Protein Interaction (PPI) Using Over-Representation Analysis (ORA)

We used ORA to perform independent assessments of GO enrichment, KEGG pathway analysis, and PPI of all DEPs in the glioma subgroups. For the annotation of the GO terms, we used the UniProt accession numbers^12^ of the Bioconductor annotation data package org.Hs.eg.db. For the annotation of KEGG data, we used the direct download from the KEGG Web site (https://www.kegg.jp/). For these analyses, we utilized the clusterProfiler^26,27^ package in R^15^ and the STRING database enrichment tool (Version 12.0).^28^

### GO Enrichment and KEGG Pathway Analysis Using Gene Set Enrichment Analysis (GSEA)

We used GSEA to perform independent assessments of both GO enrichment and KEGG pathway analysis. In our GSEA approach, we utilized two distinct outcomes: the logarithmic fold change (log_2_FC) and the signed *p*-value (which was determined based on the direction of the log_2_FC). To ensure the robustness of our findings, we only considered terms that were significantly enriched based on both the log_2_FC and the signed *p*-value outcomes.

Enriched terms were identified by using the highest and lowest normalized enrichment score values, as they contain the highest amounts of over- and underexpressed proteins, respectively.

For the annotation of the GO terms, we used the UniProt accession numbers^12^ of the Bioconductor annotation data package org.Hs.eg.db. For the annotation of KEGG data, we used a direct download from the KEGG Web site (https://www.kegg.jp/). For these analyses, we utilized the clusterProfiler^26,27^ package in R.^15^

### Correlation of DEPs with Survival of Our Cohort

To clinically validate our data, we performed survival analysis to investigate the relationship between the three most overexpressed and the three most underexpressed differentially expressed proteins (DEPs) and survival. We first split the DEP abundance into high- and low-expression groups at the median. Using the dichotomized data, we applied Kaplan–Meier survival analysis to estimate survival curves and used the log-rank test to assess differences between these groups.

Further, we performed univariable Cox proportional hazards regression using the continuous DEP abundance to quantify the association between protein expression levels and survival outcomes. For this analysis, we reported hazard ratios (HRs) along with 95% confidence intervals (CIs) to estimate the risk associated with each unit increase in protein abundance.

### Correlation of the DEPs with mRNA Expression and Survival Data from the Cancer Genome Atlas (TCGA)

To externally validate our proteomics data, we compared them with the publicly available TCGA^29^ glioma mRNA expression and survival data from a composite cohort consisting of 152 HGG and 515 LGG patients (TCGA GBMLGG) available on the GlioVis platform.^30^

### Functional Validation Using Inhibitor Response Assays

#### Dissociation of GB Tumors

Freshly resected tumor pieces were manually minced using razor blades and enzymatically dissociated at 37 °C for 30 min with 1 mg/mL collagenase type IV (Worthington Biochemical Corporation, USA) and 250 μg/mL DNase I (Roche, Switzerland) in a dissociation buffer containing HBSS (with Ca^2+^/Mg^2+^), 1% nonessential amino acids, 1 mM sodium pyruvate, 44 mM sodium bicarbonate, 25 mM HEPES, 1% GlutaMAX-I, and 1% antibiotic-antimycotic (all from Gibco, USA). The resulting cell suspensions were filtered through a 70 μm strainer and centrifuged in a 0.9 M sucrose gradient. Erythrocytes were removed using ACK lysing solution (Thermo Fisher Scientific, USA), and cell suspensions were frozen in Bambanker Standard until further use.

### Inhibitor Response Assays

To perform a functional validation of the identified targets, cells were plated at a concentration of between 3500 and 15,000 cells per well in low-adherence 384-well plates. 24 h after plating, seven inhibitors targeting the identified DEPs were selected (Table S2) and an 8- to 9-point dilution series of each compound was dispensed in four replicates using a Tecan Digital Dispenser D300e (Tecan). Drug concentrations spanned from 50 pM to 500 μM, depending on the drug. Cell viability was measured by a CellTiter-Glo 3D assay following 3 days of drug incubation, and results were normalized to untreated controls. Data analyses were performed using GraphPad Prism, applying nonlinear regression (curve fit) and the equation [inhibitor] vs normalized response (variable slope).

To assess differences in cell viability across different patients for each compound and concentration, the Kruskal–Wallis rank sum test was used as a global test for differences, followed by Dunn’s post hoc test with Bonferroni correction for pairwise comparisons. The threshold of statistical significance was set at *p* < 0.05. All statistical analyses were performed using R.

### Reference Data

Some reference data shown in this study are, in whole or in part, based upon data generated by the TCGA Research Network (https://www.cancer.gov/tcga, TCGA GBMLGG cohort),^29^ by GlioVis (http://gliovis.bioinfo.cnio.es/, TCGA GBMLGG cohort),^30^ by the STRING Consortium 2023 (https://string-db.org/),^28^ and by UniProt (https://www.uniprot.org).^12^

### Role of the Funding Sources

The study sponsors and funding sources did not have any role in the study design; in the collection, analysis, and interpretation of data; in the writing of the report; and in the decision to submit the paper for publication.

## Results

### Screening and Recruitment

A total of 122 glioma patients undergoing surgery for a diffuse glioma between July 2017 and February 2020 at the Department of Neurosurgery of the University Hospital Basel were initially screened for eligibility for this study. After confirmation of histologic and molecular diagnoses, subgroup assignment was defined by DNA methylation-based classification.^8^ We performed proteomic analysis on tumors from 86 patients. After the exclusion of four patients (GB CL_061, GB MES_072, GB MES_073, and IDHLGG_027) due to poor or missing proteomic data, 82 patients remained for final analysis.

### Baseline Characteristics

The mean age at diagnosis was significantly lower in the *IDH* mutants compared to the GB, *IDH*-wildtype subgroups (*p* < 0.001). Patients from all subgroups had a similar mean Karnofsky index (*p* = 0.642) and sex ratio (*p* = 0.692). Patients in the LGG subgroup had epilepsy in 85.3% of cases compared to lower values in the HGG subgroups (ranging from 50% to 69.2%); however, this difference did not reach statistical significance (*p* = 0.139) (Table 1). Patients with LGG had a significantly longer median survival than those with HGG. *IDH* HGG did show a significantly longer median survival compared to the other HGG subgroups, whereas there was no difference between the median survival of the three GB, *IDH*-wildtype subgroups (Figure S1).

**Table 1:** Baseline Characteristics of the Glioma Subgroups^a^

Baseline Characteristics	Diffuse LGG (*N* = 34)	*IDH* HGG (*N* = 12)	GB PN (*N* = 9)	GB CL (*N* = 14)	GB MES (*N* = 13)	*p*-Value
Male sex (%)	24/34 (70.6%)	6/12 (50%)	7/9 (77.8%)	9/14 (64.3%)	8/13 (61.5%)	0.692^b^
Epilepsy (%)	29/34 (85.3%)	6/12 (50%)	6/9 (66.7%)	9/14 (64.3%)	9/13 (69.2%)	0.139^b^
Median age (range)	44.5 (20–79)	38 (27–73)	60 (55–78)	66 (48–76)	59 (52–79)	<0.001^c^
Mean Karnofsky index (range)	80 (70–100)	80 (70–100)	80 (70–90)	80 (60–90)	80 (70–90)	0.642^c^
Surgery type (GTR: gross total resection, STR: subtotal resection)	21 GTR	5 GTR	5 GTR	6 GTR	5 GTR	0.496^b^
	13 STR	7 STR	4 STR	8 STR	7 STR	
					1 biopsy	
Tumor side	16 right sided	5 right sided	5 right sided	11 right sided	4 right sided	0.379^b^
	15 left sided	6 left sided	4 left sided	3 left sided	7 left sided	
	3 bilateral	1 bilateral			2 bilateral	
Most common affected lobe	23/34 frontal	8/12 frontal	6/9 temporal	7/14 parietal	7/13 temporal	n/a

a bThe two low grade glioma subgroups are pooled as one group (diffuse LGG).

b Fisher’s exact test.

c Kruskal–Wallis rank sum test.

The *IDH*-mutated tumors were most commonly located in the frontal lobe, whereas GB PN and GB MES were most commonly found in the temporal lobe, and GB CL in the parietal lobe. 21/34 LGG (61.7%), 5/12 *IDH* HGG (41.7%), 5/9 GB PN (55.6%), 6/14 GB CL (42.9%), and 5/13 GB MES (38.5%) underwent gross total resection. 2/82 patients had chemotherapy, and only 1/82 patients had radiotherapy prior to the surgery where the sample for the proteomic analysis was taken. 46/82 patients had been given steroids prior to surgery. As expected, this rate was higher in the HGG subgroup (35/48 patients; 72.9%) than in the LGG subgroup (11/34 patients; 32.4%) (Table S3). Neither treatment prior to surgery (chemotherapy, radiotherapy, steroids) nor confounding factors (age, epilepsy) were significantly associated with any of the DEPs, although sex impacted the expression of Y-chromosome-encoded adenosine triphosphate (ATP)-dependent RNA helicase DDX3Y (DDX3Y).

Additional collected data comprised local genetic imbalances, such as *epidermal growth factor receptor (EGFR)* amplification (in addition to chromosome 7 triploidy) and *cyclin-dependent kinase inhibitor 2A* (*CDKN2A*) homozygous deletion statuses; *telomerase reverse transcriptase* (*TERT*) promoter mutations; and TP53 immunopositivity (as an indicator of possible *TP53* mutation when higher than 20%),^31^ and hemizygous codeletions at the 1p and 19q chromosome arms, as well as methylomic data. Taken together, these data converged to consistent definitions of the subgroups of our cohort (Table S3).

By the introduction of HG2A, PTPRC/CD45 (reflecting immune cell contents in tumors), and SEPT3 (neuron contents) as non-neoplastic cell markers into the multivariate model, 464 proteins that were significantly different between at least two subgroups before were removed from the model, and 137 proteins were added to the total amount of DEPs between the subgroups.

### Proteomic Profiles Primarily Segregate LGG from HGG, and *IDH* Mutant from *IDH*-Wildtype Tumors

After the exclusion of samples with missing protein data, 5057 common proteins were identified and quantified in each tumor sample. Profiles of DEPs distinguished the *IDH*-mutated subgroups (LGG and *IDH* HGG) from the three *GB, IDH*-wildtype subgroups (Figure 1a), while PCA analysis and UMAP both showed a main separation driven by tumor WHO grade, followed by *IDH* mutation status (Figure 2a,b). The largest differences in DEP numbers were observed between GB CL and LGG (162 DEPs), followed by GB PN and LGG (144 DEPs), GB MES and LGG (28 DEPs), and lastly *IDH* HGG and LGG (1 DEP) (Figure 1b). We did not observe DEPs that were significantly overexpressed in all four HGG subgroups (Figure 1c).

**Figure 1:**
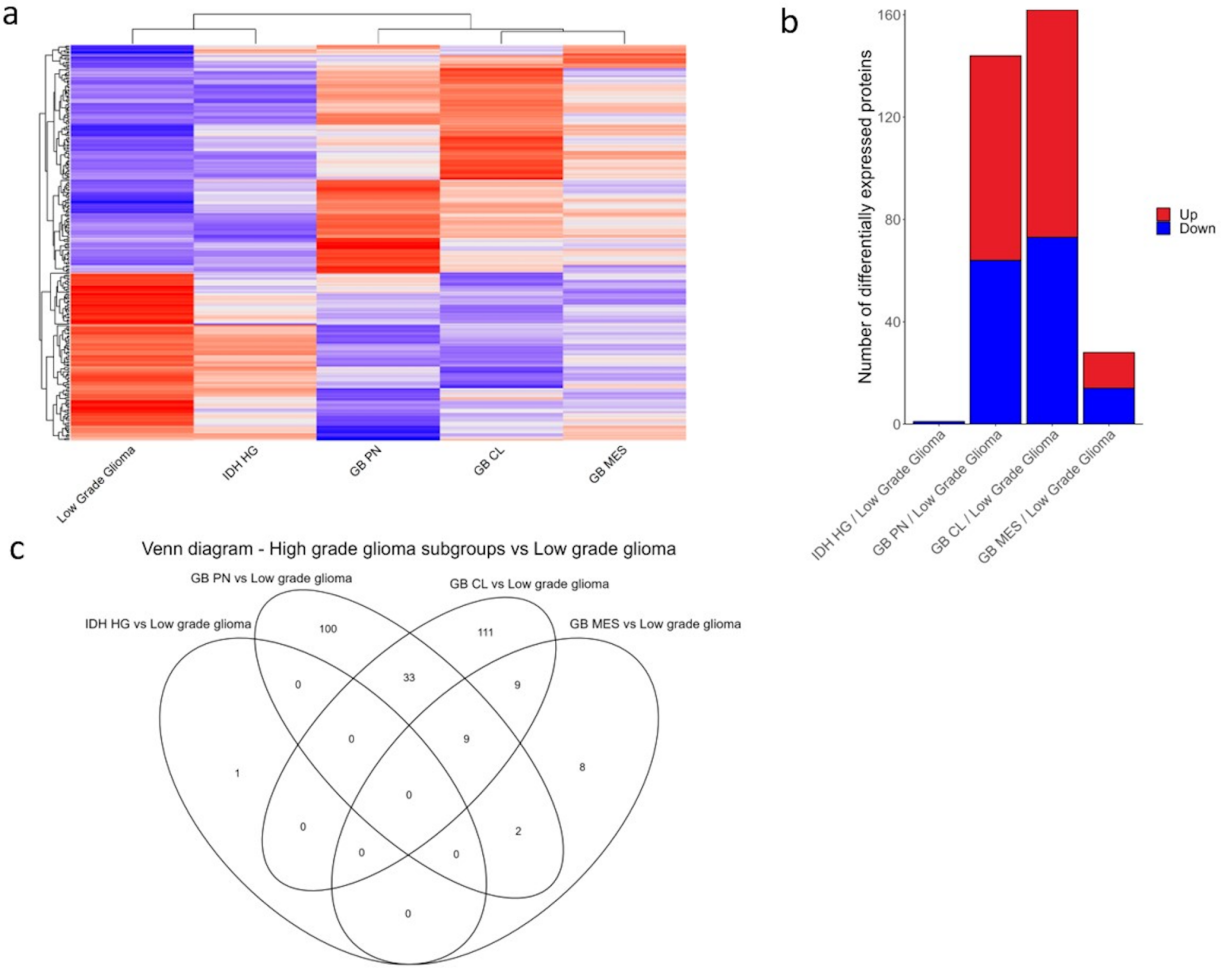
Proteomic profiles segregate LGG from HGG and *IDH-*mutant from *IDH-*wildtype tumors. (a) Heatmap of abundance values of DEPs between LGG and HGG subgroups. Overexpressed proteins are shown in red, and underexpressed proteins are shown in blue (this applies also to Figure (b)). (b) Number of DEPs if comparing the four HGG subgroups to the pooled LGG subgroups. (c) Venn diagram showing the DEPs that are common between the LGG and each HGG subgroups. There was no common protein significantly differentially expressed between LGG and all four HGG subgroups.

**Figure 2:**
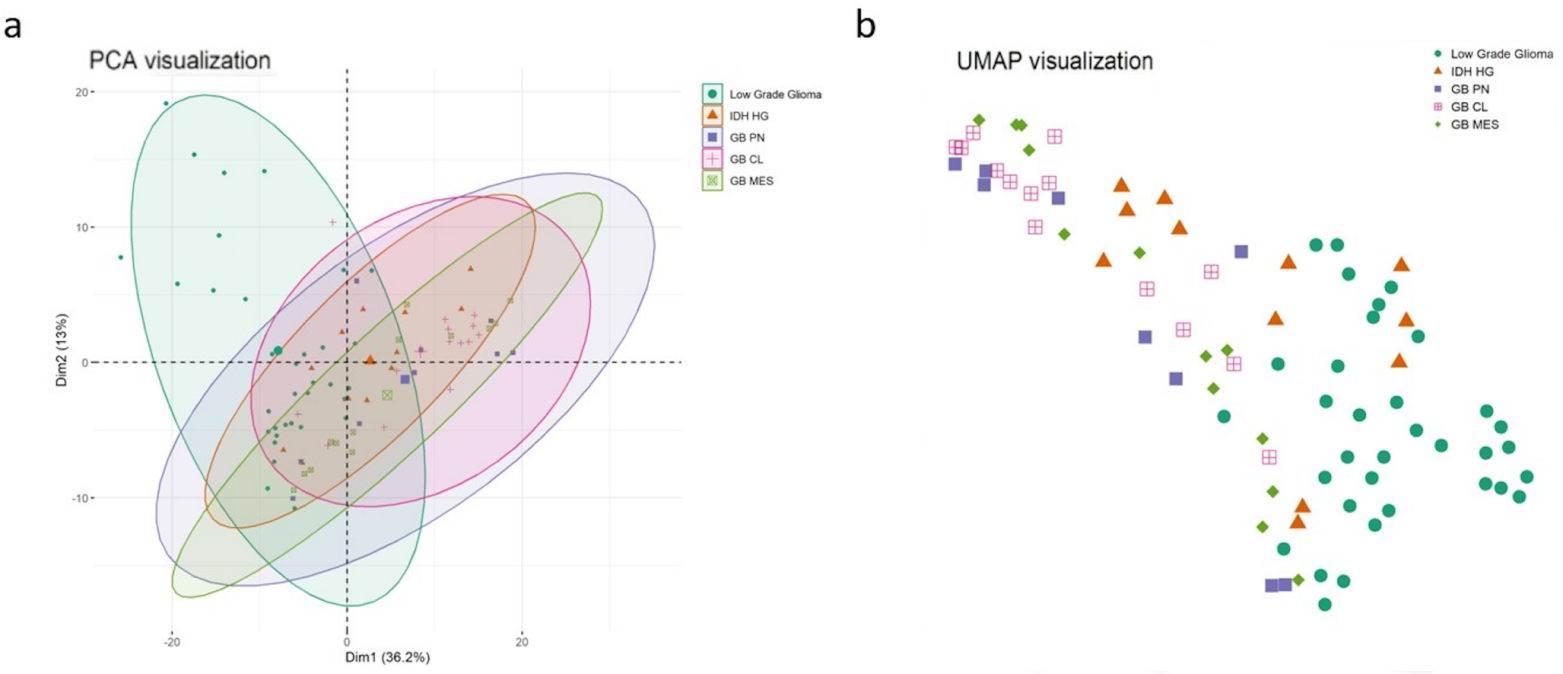
Dimensionality reduction using PCA and UMAP shows the segregation of LGG from HGG. (a) *IDH* status and tumor grade drive separation in the PCA. (b) UMAP showing the differential clustering of the DEPs of the LGG and all four HGG subgroups according to tumor grade and *IDH* status.

### GB PN Displayed Enrichment in Functions Important for Cell Proliferation

Among the 144 DEPs between GB PN and pooled LGG (Figure 3a), 80 proteins were overexpressed in GB PN, and 64 were underexpressed in GB PN (compared to LGG) (Tables S4 and S5).

**Figure 3:**
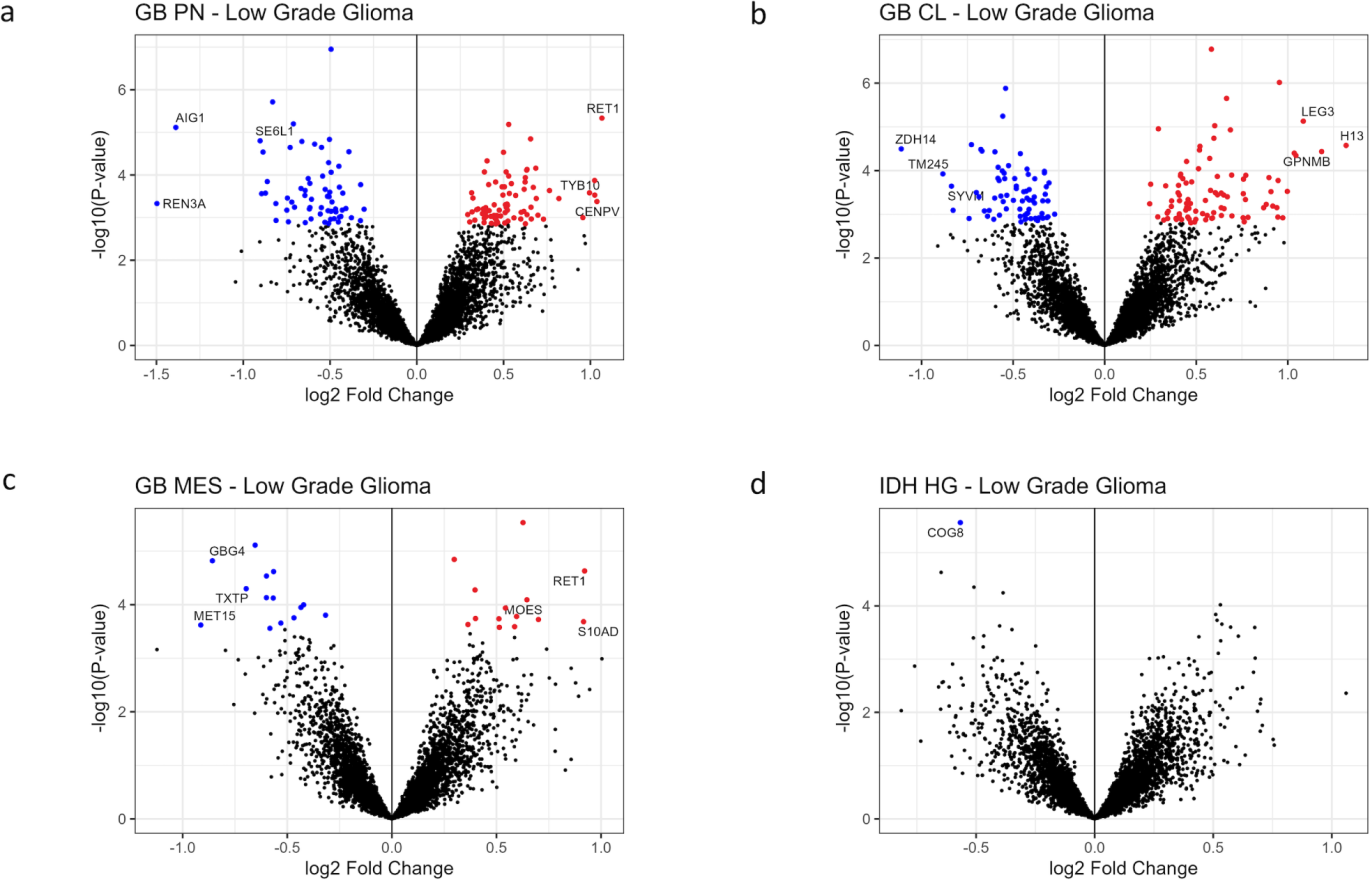
Volcano plots of DEPs between each HGG subgroup and LGG. (a) Volcano plot of 144 DEPs between the GB PN and LGG; red and blue dots indicate significantly over- and underexpressed proteins, respectively (this applies also to Figures (b–d)). (b) Volcano plot of 162 DEPs between the GB CL and LGG. (c) Volcano plot of 28 DEPs between the GB MES and LGG. (d) Volcano plot of 1 DEP between the *IDH* HGG and LGG.

RalA-binding protein 1 (RBP1), centromere protein V (CENPV), and thymosin beta 10 (TYB10) were the proteins showing the highest log_2_FC values in GB PN (compared to LGG). Translation and cell cycle/telomere regulation were dominantly associated with overexpressed DEPs in GB PN, and the most enriched GO cellular compartment was the ribosome (Tables 2 and S6 and Figure 4a).

**Table 2:** Functional Enrichment Detected Using the STRING Functional Enrichment Tool^28^ Including GO and KEGG Annotation among the Over- and Underexpressed Proteins in the HGG Subgroups (Compared to LGG)^a^

HGG subgroup (compared to LGG)	Pathways and functions containing over- and underexpressed DEP, respectively	
GB PN	Containing overexpressed DEP	Translation (ORA and GSEA)
		Cell cycle/telomere regulation (only GSEA)
	Containing underexpressed DEP	Mitochondrial organization
		Lipid metabolism
		Pentose phosphate pathway
GB CL	Containing overexpressed DEP	Cell adhesion
		Actin cytoskeleton
		Cell death/apoptosis regulation
	Containing underexpressed DEP	Mitochondrial organization
		Lipid metabolism
		Protein metabolism
GB MES	Containing overexpressed DEP	Mitochondrial ATP synthesis (ORA and GSEA)
		Actin cytoskeleton (only GSEA)
	Containing underexpressed DEP	Translation (only GSEA)
		Lipid metabolism (only GSEA)
*IDH* HGG	Containing overexpressed DEP	Mitochondrial ATP synthesis (only GSEA)
	Containing underexpressed DEP	Golgi organization (ORA and GSEA)
		Translation (only GSEA)

a If not otherwise specified, enrichment was found in over-representation analysis (ORA) and gene set enrichment analysis (GSEA).

**Figure 4:**
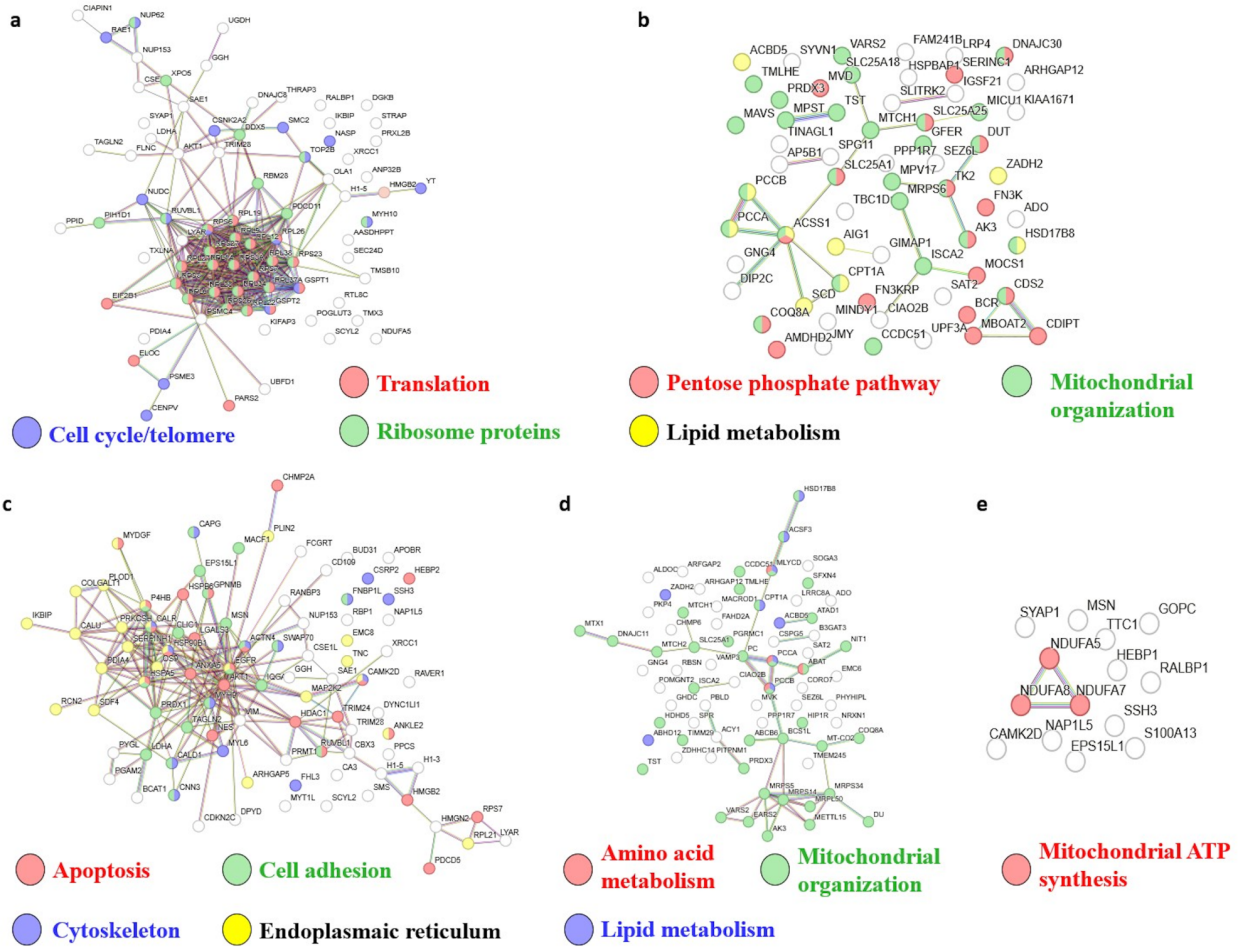
ORA (over-representation analysis) of the DEPs over- and underexpressed in the GB, *IDH*-wildtype subgroups (compared to LGG) using the STRING functional enrichment tool (KEGG, GO, and PPI). (a) Translation (network nodes in red), ribosome proteins (green), and cell cycle/telomere regulation (blue) were dominantly increased in the 80 DEPs overexpressed in GB PN. The most enriched GO cellular compartment was the ribosome. (b) Functions involved in mitochondrial organization (network nodes in green), lipid metabolism (yellow), and pentose phosphate pathway (red) were enriched in the 64 DEPs underexpressed in GB PN. The most enriched GO cellular compartment was the mitochondrion (green). (c) Functional enrichment was seen in cell adhesion (network nodes in green), actin cytoskeleton (blue), and cell death/apoptosis (red) among the 89 DEPs overexpressed in GB CL. The most enriched GO cellular compartment was the endoplasmic reticulum (yellow). (d) Functional enrichment was seen in mitochondrial organization (network nodes in green), lipid metabolism (blue), and amino acid metabolism (red) among the 73 proteins underexpressed in GB CL. The most enriched GO cellular compartment was the mitochondrion (green). (e) Functional enrichment was seen in mitochondrial ATP synthesis (red) among the 14 overexpressed DEPs in GB MES. Note: results for the 14 DEPs underexpressed in GB MES are not shown, as these were seen only in GSEA (and not in ORA).

Regulator of nonsense transcripts 3A (REN3A), androgen-induced gene 1 protein (AIG1), and seizure 6-like protein (SE6L1) showed the highest log_2_FC values in LGG (compared to GB PN). Functions involved in mitochondrial organization, lipid metabolism, and pentose phosphate pathway appeared enriched among underexpressed DEPs, and the mitochondrion was the most enriched GO cellular compartment among the underexpressed DEPs in GB PN (compared to LGG) (Tables 2 and S6 and Figure 4b).

### Overexpressed DEPs in GB CL Exhibited Functions Required for Cell Migration and Invasion

Among the 162 DEPs between GB CL and pooled LGG (Figure 3b), 89 proteins were overexpressed in GB CL, and 73 proteins were underexpressed in GB CL (compared to LGG) (Tables S7 and S8).

Histone H1.3 (H13), glycoprotein NMB (GPNMB), and galectin-3 (LEG3) were the proteins that showed the highest log_2_FC values in GB CL (compared to those of LGG). In GB CL, functional enrichment was seen in cell adhesion, actin cytoskeleton, and cell death/apoptosis. The most enriched GO cellular compartment was the endoplasmic reticulum (Tables 2 and S6 and Figure 4c).

Palmitoyltransferase ZDHHC14 (ZDH14), transmembrane protein 245 (TM245), and valine-tRNA ligase, mitochondrial (SYVM) showed the highest log_2_FC values in LGG (compared to those of GB CL). DEPs underexpressed in GB CL did show functional enrichment in mitochondrial organization, and lipid as well as protein metabolism. The mitochondrion was the most common GO cellular compartment among the underexpressed DEPs in GB CL (Tables 2 and S6 and Figure 4d).

### GB MES Displayed Signs of Activated Metabolism

Among the 28 DEPs between GB MES and pooled LGG (Figure 3c), 14 proteins were overexpressed in GB MES, and 14 were underexpressed in GB MES (compared to LGG) (Tables S9 and S10).

RBP1, protein S100-A13 (S10AD), and moesin (MOES) were the proteins that showed the highest log_2_FC values in GB MES (compared to LGG). Overexpressed DEPs in GB MES showed functional enrichment in mitochondrial ATP synthesis and the actin cytoskeleton (Tables 2 and S6 and Figure 4e).

12S rRNA N4-methylcytidine methyltransferase (MET15), guanine nucleotide-binding protein G­(I)/G­(S)/G­(O) subunit gamma-4 (GBG4), and tricarboxylate transport protein mitochondrial (TXTP) did show the highest log_2_FC values in LGG (compared to GB MES). Underexpressed DEPs in GB MES (compared to LGG) did show functional enrichment in translation and lipid metabolism (Table 2).

### *IDH* HGG and LGG Were Characterized by Similar Proteomic Features

Only one protein, Conserved Oligomeric Golgi Complex Subunit 8 (COG8), was significantly overexpressed in pooled LGG compared to *IDH* HGG, with a log_2_FC of 0.56 (Figure 3d, Tables S11 and S12).

Mitochondrial ATP synthesis was associated with overexpressed DEPs in *IDH* HGG in the GSEA, while the underexpressed DEPs in *IDH* HGG were associated with Golgi organization (Tables 2 and S6).

### Different Protein Expression between HGG Subgroups

Individual comparisons revealed 22 DEPs between GB CL and *IDH* HGG (Table S13), whereas there were no DEPs between the other individual HGG subgroups. Ras GTPase-activating-like protein IQGAP1 (IQGA1) was the sole overexpressed protein in GB CL compared with the other pooled HGG subgroups (Table S14), a protein that plays a crucial role in regulating the dynamics and assembly of the actin cytoskeleton.

### The Majority of the Most Over- and Underexpressed DEPs Were Significantly Associated with Overall Survival

We analyzed the three most overexpressed and three most underexpressed DEPs from each subgroup in relation to overall survival within our cohort. High expression of TYB10, H13, GPNMB, LEG3, S10AD, and MOES was significantly associated with shorter overall survival, whereas high expression of SE6L1, AIG1, REN3A, SYVM, TM245, TXTP, GBG4, and MET15 was significantly associated with longer overall survival (Figure S2). However, two of the most underexpressed proteins (ZDH14 (*p* = 0.18), and COG8 (*p* = 0.74)) and two of the strongest overexpressed proteins (RBP1 (*p* = 0.89) and CENPV (*p* = 0.86)) did not show a statistically significant association with survival (Figure S2 and Table S15).

### Our Results Showed Good Correlation with Transcriptomic and Survival Data from TCGA

We compared our proteomics data with the publicly available TCGA^29^ glioma mRNA expression and survival data from a composite cohort consisting of 152 HGG and 515 LGG patients (TCGA GBMLGG) available on the GlioVis platform.^30^ Most of the proteins significantly overexpressed in our HGG subgroups showed higher mRNA levels in GB compared to LGG, and vice versa for the proteins that were underexpressed in the HGG subgroups (Figures S3–S6). Higher mRNA expression levels of all the proteins overexpressed in our HGG subgroups were associated with worse survival (compared to lower mRNA expression levels) (Figures S2–S4). However, the TCGA survival data was less consistent for the mRNA expression coding for proteins that were significantly underexpressed in our HGG subgroups (Figures S3–S6).

### Functional Validation with Inhibition of the Most Overexpressed DEPs Led to Reduced Cell Viability at Higher Concentrations That Was Different between GB, *IDH*-Wildtype Subgroups

To assess the functional relevance of the DEPs in each subgroup and their potential subgroup specificity, we tested the efficacy of protein inhibitors in a viability assay using patient-derived, noncultured, subtyped^8^ GB single-cell suspensions, with a sample from each subgroup and one sample defined as a “mixed” sample expressing GB PN and CL characteristics. Inhibition of those most overexpressed DEPs reduced cell viability in a partly patient-specific manner, potentially depending on the GB subgroup.

Inhibition of LEG3 (overexpressed in GB CL) using GB1107 ^32^ resulted in reduced cell viability in samples of all subgroups, most pronounced in the GB CL sample (significant difference to the GB MES sample at 500 μM). Thus, overexpression of LEG3 in GB CL might lead to higher susceptibility against inhibition (Figure S7).

Targeting cells that express GPNMB (overexpressed in GB CL) using the antibody–drug conjugate Glembatumumab vedotin^33,34^ resulted in only a moderate cell viability reduction across all samples, even at the highest concentrations. However, it impacted the mixed sample at higher concentrations significantly compared to the GB PN sample (Figure S8).

Increasing concentrations of the histone deacetylase inhibitor Vorinostat^35^ (inhibiting H13 that is overexpressed in GB CL) led to stepwise reduced cell viability in samples of all subgroups. The GB CL sample preserved the highest levels of cell viability up to a dose range of 100 μM, but the GB MES sample outperformed the GB CL sample at 500 μM. The cell viability of the GB PN sample was stable at lower concentrations but decreased rapidly at higher concentrations. Hence, the overexpression of H13 might lead to more robustness against inhibition (Figure S9).

Inhibition of CENPV (overexpressed in GB PN) using GSK-923295 ^36,37^ reduced cell viability sharply in samples of all subgroups at dose ranges between 50 and 100 μM. At 50 μM, significant differences between the mixed sample and the GB CL and GB PN samples could be observed. However, GSK-923295 specifically inhibits CENPE, and thus the effect on CENPV could be unspecific (Figure S10).

Inhibition of S10AD (overexpressed in GB MES) using Amlexanox^38^ reduced cell viability in samples of all subgroups at higher concentrations and most in the GB MES sample, even though without a statistically significant difference at 500 μM (Figure S11).

Inhibition of MOES (overexpressed in GB MES) was performed using NSC668394 ^39^ and Fasudil HCl^40^ (indirect inhibitor). Inhibition of MOES with NSC668394 led to a sudden reduction in cell viability at concentration ranges of 50 and 100 μM, with the least impact in the GB MES sample at 50 μM (Figure S12). Indirect inhibition of MOES with Fasudil HCl led to reduced cell viability in samples of all subgroups at increasing concentrations but also less in the GB MES sample at 500 μM (Figure S13). Hence, the overexpression of MOES in GB MES might be protective against inhibition.

## Discussion

### Definition of Glioma Subgroups and Introduction of Non-neoplastic Cell Markers

We present a primarily descriptive comparative proteomic analysis of glioma subgroups, accurately defined using methylomic classification.^8^ Our findings demonstrate that glioma grade and *IDH* mutation status are the major discriminators for protein expression profiles across glioma subgroups. Using high-quality proteomic data collected intraoperatively, we identified the most overexpressed DEPs within each subgroup and correlated their expression with survival outcomes in our glioma cohort, as well as with mRNA expression and survival data from the TCGA cohort.

Distinct patterns of pathways and biological functions emerged across the three GB, IDH-wildtype subtypes:

GB PN was characterized by enrichment in translation processes and cell cycle/telomere regulation, supporting proliferative capacity.
GB CL showed upregulation of proteins involved in actin cytoskeleton organization, cell adhesion, and cell death/apoptosis regulation, consistent with migratory and invasive behavior.
GB MES exhibited elevated mitochondrial ATP synthesis activity, highlighting metabolic activity.

For each glioma subgroup, we proposed potential novel biomarkers and validated their associations with survival in both our cohort and through mRNA expression and survival analyses from TCGA data. Additionally, exploratory functional validation using inhibitor response assays on dissociated tumor cells suggested that targeting the most overexpressed DEPs reduced cell viability in a dose-dependent manner. However, given that functional assays were conducted on only one sample per subgroup, these results should be interpreted as preliminary and hypothesis-generating rather than definitive and conclusive.

### The GB, *IDH*-Wildtype Subgroup Definition and Its Evolution over Time

An early proteomic study on GB in 2009 ^5^ delineated three subclasses of GB characterized by distinct patterns of protein expression and activation within glioma-relevant signaling pathways. One subgroup exhibited a predominance of platelet-derived growth factor receptor (PDGFR) activation (reminiscent of GB PN), another displayed a predominance of EGFR activation (reminiscent of GB CL), and a third demonstrated loss of the rat sarcoma (RAS) regulator neurofibromin 1 (NF1) (reminiscent of GB MES).^5^ The understanding of the GB subgroups and heterogeneity was refined in 2019 as Neftel et al. described GB to exist in four different cellular states.^9^ They, as well as previous studies, have shown that various states or subtypes do coexist within the same GB.^9,41,42^ A shift in the predominant state or subtype occurs in patients^43^ and can be induced experimentally.^4^ Tumor recurrences in GB typically maintain the same state or subtype domination, although studies have shown that chemoradiation holds the potential to induce a transition from proneural to mesenchymal (PMT), akin to the epithelial–mesenchymal transition (EMT) observed in carcinomas, which is associated with therapy resistance.^44^ The mechanisms underlying PMT remain poorly understood; however, the tumor microenvironment appears to play a crucial role.^45^ In cancer, the evolutionary trajectory and coexistence of distinct subpopulations of cancer cells within the same tumor likely underlie therapy failure, the development of treatment resistance, and the eventual recurrence of the malignancy.^46^ Therefore, comprehending the composition of GB subpopulations is crucial for effective disease management and targeted therapy.

### Results of Our Study in Relation to Recent Proteomic Studies in Glioma

Zhang et al. conducted proteomic profiling on 343 glioma tumor samples and 53 normal brain tissues, identifying two distinct proteomic subtypes: (1) metabolic neural subgroup: characterized by enriched metabolic enzymes and neurotransmitter receptor proteins and (2) immune subgroup: marked by upregulation of immune and inflammatory proteins. The research highlighted significant differences in prognosis and the tumor microenvironment between these subgroups. Notably, enzymes DPYD and TYMP were identified as potential prognostic biomarkers, suggesting nucleotide metabolism as a promising therapeutic target.^47^

Another study performed in 2023 conducted proteogenomic profiling on 213 glioma tumors, uncovering three proteomic subgroups: (1) S-Ne (neural): associated with neural characteristics, (2) S-Pf (proliferative): linked to cell proliferation pathways, and (3) S-Im (immune): characterized by immune-related features. This study also identified that EGFR alterations could lead to tumor proliferation through ERK5-mediated nucleotide synthesis. Additionally, higher EGFR alteration frequencies were associated with increased expression of immune checkpoint proteins PD-L1 and CD70 in T-cell-infiltrated tumors.^48^

Shen et al. introduced a spatially multidimensional comparative proteomics strategy using glioma samples. By analyzing protein expression across different tumor regions, the research provided insights into the heterogeneity of gliomas. The approach allowed for the identification of region-specific protein expression patterns, contributing to a better understanding of glioma biology, heterogeneity, and potential therapeutic targets.^49^

Bader et al. conducted proteomic and phosphoproteomic analyses on 42 glioma biopsies with known *IDH* and 1*p*/19q codeletion statuses. The study found that *IDH* mutant gliomas could be divided into two distinct entities independent of 1p/19q codeletion status: one subgroup exhibited accumulation of cancer drivers and a lower abundance of mitochondrial respiratory chain proteins, potentially leading to shorter survival. These findings suggest that proteomic profiling can provide additional stratification beyond genetic markers, offering new avenues for targeted therapies.^50^ Duhamel et al. presented, in 2022, a spatial proteomic characterization in clinical samples of glioblastoma. They were able to identify three subregions with differing protein expression (Group A: proteins associated with neurogenesis, Group B: immune system activation, and Group C: tumorigenesis) with high intratumoral heterogeneity.^51^ Lam et al. leveraged mass spectrometry in 2022 to spatially align abundance levels of 4794 proteins to distinct histologic tumor niches across 20 GB patients. They defined two distinct proteogenomic programs, MYC- and KRAS-axis hereon, that cooperate with hypoxia to produce a tridimensional model of intratumoral heterogeneity. Interestingly, high KRAS target activity was associated with invasion and epithelial-to-mesenchymal transition processes (similar to our results in the GB CL and GB MES subgroups), whereas samples enriched for the MYC axis were associated with cell cycle progression (similar to our results in the GB PN subgroup).^52^

To conclude, several studies have recently attempted at defining the proteome, comparing it to the transcriptome, and understanding the heterogeneity of glial tumors, especially GB. Several studies showed that proteomics can subclassify gliomas even with a common genetic background. Proteomic-based classification of gliomas created subtypes with different prognoses, responses to therapy, and possibly eligibility for immunotherapy or metabolic interventions. Each of these studies used a slightly different approach, and all of those are of complementary value. Our study is different from those studies as we (1) also included LGG and *IDH*-mutated HGG subgroups, not only; (2) used methylomic classification^8^ to obtain a sharp subgroup definition before proteomic analysis; (3) included factors potentially impacting protein expression levels, such as age, sex, treatments (chemotherapy, radiotherapy, steroids), and epilepsy as confounding factors in the model; and (4) included the use of “non-neoplastic protein markers” (HG2A, PTPRC, and SEPT3), primarily associated with tumor-associated immune cells and neurons, to estimate their proportions in the tumor samples.

We provide diverse functions and pathways enriched with overexpressed DEPs for each GB, *IDH*-wildtype subgroup: translation and cell cycle/telomere regulation in GB PN (linked to proliferation); actin cytoskeleton, cell adhesion, and apoptosis regulation in GB CL (linked to migration and invasion); and mitochondrial ATP synthesis in GB MES (linked to metabolism), a novel finding that has not been reported in this way previously.

For all of these reasons, our study adds complementary value and substantially amends the current knowledge of the proteome of glial tumors.

### Pathway Analyses Revealed Different Biological Processes and Molecular Functions in Each Glioma Subgroup

The major discriminators for protein expression levels in our study were glioma grade and *IDH* mutation status, as shown previously in a study comparing glioma subgroups of various grades and *IDH* mutation status.^53^

Many of the overexpressed DEPs in the GB PN were associated with functions related to translation and protein synthesis. Several studies have previously identified enrichment of DNA replication and cell cycle gene transcripts in the GB PN subtype.^54^ Sustaining telomere length, either via increased *TERT* expression resulting from *TERT* promoter mutations or through alternative lengthening of telomeres (ALT) resulting from *alpha-thalassemia/mental retardation X-linked* (*ATRX*) mutation, appears to be a crucial aspect of GB development.^55^ A study from 2010 showed that GB patients with tumors with ALT had longer survival, independent of age, surgery, and other treatments. This could be attributed to the fact that most ALT+ tumors are less aggressive GB PN subtypes.^56^

The “regulation of the actin cytoskeleton” emerged as a prominent function among the proteins significantly overexpressed in GB CL. The dynamic processes of GB cell migration and invasion necessitate the restructuring of the actin cytoskeleton to alter cell shape and propel cell movement. Recent research suggests that remodeling of the actin cytoskeleton might enable cancer cell resistance to antitumor immunity.^57^ Interestingly, several proteins linked with invasion were significantly overexpressed in GB CL: MOES, EGFR, annexin A5 (ANXA5), vimentin (VIM), tenascin C (TENA), and cysteine- and glycine-rich protein 2 (CSRP2). Elevated MOES expression in GB cells leads to increased cell proliferation, invasion, and migration in vitro and in vivo.^58^ Several studies have shown that EGFR, ANXA5, VIM, and TENA are associated with unfavorable outcomes in GB.^5,6,59–61^

Another enriched function among the DEPs overexpressed in GB CL is the regulation of apoptosis and cell death. Among the mediators of these processes are EGFR, ANXA5, LEG3, GPNMB, nestin (NES), heat shock protein beta-6 (HSPB6), and transcription intermediary factor 1-alpha (TRIM24). The phosphatidylinositol-3 kinase (PI3K)/protein kinase B (AKT)/mammalian target of rapamycin (mTOR) pathway, downstream of the receptor tyrosine kinase pathway to which EGFR belongs, plays a pivotal role in cell survival, proliferation, motility, angiogenesis, and apoptosis, and is deregulated in approximately 80% of all GB cases.^62^ Phosphatase and TENsin homologue (PTEN) counteracts the PI3K/AKT/mTOR pathway, and its loss is a hallmark of GB. *EGFR* amplification and *PTEN* loss particularly co-occur in GB.^63^ IQGA1, the only protein significantly overexpressed in GB CL compared to the pool of other HGG, is involved in cell adhesion and migration of glioma cells, and its expression correlates with poor tumor grade and survival.^64^ It is a known scaffold protein in glioma stem cell niches.^65^

Citrate synthase and oxidative phosphorylation complexes gradually decrease during progression from LGG to HGG.^66^ The S10AD overexpression we found in GB MES compared to LGG refines the S10AD overexpression previously observed in HGG, correlating with tumor grade and microvessel density.^67^

### Strengths and Limitations of Our Study

This study provides comprehensive proteomic insights by analyzing and quantifying 5057 proteins for each tumor, with only minimal missing values per protein. The strengths of our study can be seen in the prospective sample collection and sharp subgroup definition before proteomic analysis using methylomic classification.

Gliomas are heterogeneous tumors containing not only neoplastic cells but also a network of infiltrating immune and other cell types. Thus, the observed protein level variations may also reflect tumor-associated cell composition, which varies from one glioma subtype to another^17^ and whose proportion can make up to 40%^55^ with the highest values of macrophage and microglia infiltration in GB MES.^17^ In addition, within each subtype collection, the biopsy cell type composition potentially varies from one resection event to another. To exclude false-positive proteins mainly associated with immune cells and neurons, we introduced the markers HG2A and PTPRC (CD45) for immune cells and SEPT3 for neurons into our model as “non-neoplastic cell markers”. However, this offers only an approximation of reality and possibly obviates the detection of interesting results, as there were many DEPs between *IDH* HGG and LGG in the uncorrected model (results not shown), and only one DEP remained after accounting for these confounders. Additionally, we did not use further validation, such as immunohistochemistry, as this was not the scope of the study. While CD45 has been utilized as a marker in proteomic studies before and has shown good correlation with CD45 immunohistochemistry,^68^ there is limited data available regarding the use of SEPT3 and HG2A as biomarkers in proteomic and other studies.

Access to true non-neoplastic brain tissue is extremely limited in neuro-oncology. This is the reason we have not included non-neoplastic controls in our study. However, validation of our results in normal tissue is critical; therefore, further validation studies are needed to confirm our findings.

Exploratory functional validation was performed by using inhibitor response assays. Patient-derived, noncultured, subgroup-defined samples were used for this analysis. These assays showed dose-dependent responses in most of the samples and trended toward the identification of patient- and potentially subgroup-specific responses in a subset of the assessed signature protein inhibitors. There were specific inhibitors available for some of the proteins, and for others, we had to use more broad or indirect inhibition of protein effects or associated pathways. As the growth of these cells in vitro is not something that is easily achieved, and only very recent samples could be used, just one sample per subgroup was available for this analysis. Moreover, the results obtained at very high inhibitor concentrations must be viewed with caution due to potential off-target effects. Further, while we have information about the molecular alterations of the tumors, we did not characterize the in vitro cell cultures derived from these tumors with regard to their protein expression. Thus, it is not possible to draw meaningful conclusions from the observed results, and they can be seen as exploratory and hypothesis-generating rather than confirmatory. Continuing validation is needed in larger cohorts of subtyped single-cell suspensions and more stringent dose–response curves assessing the subtype-defining DEPs from our data set.

The constrained sample sizes within the subgroups and the imbalances of samples between subgroups may have impacted the statistical power of differential protein expression between groups. Additionally, this weakens the generalizability of the results.

## Conclusion

Quantitative proteomic analysis across glioma subgroups highlighted tumor grade and *IDH* mutation status as the primary determinants of protein-level variations. Delving deeper into the expression profiles of the three GB, *IDH*-wildtype subgroups revealed distinct patterns of overexpressed proteins associated with glioma progression-related pathways: cell proliferation in GB PN, cell migration and invasion in GB CL, and metabolism in GB MES.

This primarily descriptive study provides valuable insights into tumor subgroup metabolism and potential biomarkers for further experimental testing. A better understanding of the metabolic dynamics of GB, its heterogeneity, and subgroups is crucial for informing disease management strategies and facilitating future targeted therapy for this lethal malignancy. While our results highlight promising subgroup-specific differences, they are rather hypothesis-generating than conclusive, and further studies on larger cohorts are necessary to validate these findings and assess their clinical relevance.

## Supplementary Material





## Data Availability

The generated raw proteomic glioma data inventory was uploaded to the public MassIVE database repository (project accession number: PXD041647, https://massive.ucsd.edu/ProteoSAFe/private-dataset.jsp?task=07d1eb41382b48dd8dd03b77af856077), and the raw data from the inhibitor response assays was uploaded to Mendeley Data (10.17632/msr3c3rnt6.1). The R codes from the scripts that were used for data analysis were uploaded to https://github.com/SORC-Basel/proteomics-idhwt-glioblastoma. A preprint of this manuscript was uploaded to medRxiv (MEDRXIV/2025/323141). The abstract graphic was designed using BioRender and can be accessed here: https://BioRender.com/vzhp24s.

## Notes

Kaplan–Meier curve showing the overall survival of the 6 glioma subgroups (Figure S1); association of the three most overexpressed and three most underexpressed DEPs from each subgroup with overall survival (Figure S2); TCGA data using the TCGA GBM-LGG cohort showing the mRNA expression levels of the DEPs most over- and underexpressed in GB PN, and association of high and low mRNA levels of these DEPs with survival (Figure S3); TCGA data using the TCGA GBM-LGG cohort showing the mRNA expression levels of the DEPs most over- and underexpressed in GB CL, and association of high and low mRNA levels of these DEPs with survival (Figure S4); TCGA data using the TCGA GBM-LGG cohort showing the mRNA expression levels of the DEPs most over- and underexpressed in GB MES compared to LGG, and association of high and low mRNA levels of these DEPs with survival (Figure S5); TCGA data using the TCGA GBM-LGG cohort showing the mRNA expression level of the only DEP significantly underexpressed in IDH HGG compared to LGG, and association of high and low mRNA levels of this DEP with survival (Figure S6); inhibitor response assay using GB1107 targeting LEG3 (Figure S7); inhibitor response assay using Glembatumumab vedotin targeting GPNMB (Figure S8); inhibitor response assay using Vorinostat targeting H13 (Figure S9); inhibitor response assay using GSK-923295 targeting CENPV (Figure S10); inhibitor response assay using Amlexanox targeting S10AD (Figure S11); inhibitor response assay using NSC668394 targeting MOES (Figure S12); inhibitor response assay using Fasudil HCl targeting MOES (Figure S13) (PDF)
Experimental design of the TMT proteomics experiment (Table S1); differentially expressed proteins, inhibitors, and suppliers (Table S2); detailed demographic, clinical, treatment, survival, and molecular genetic data listed for each tumor sample (Table S3); differentially expressed proteins between GB PN and LGG (Table S4); differentially expressed proteins with the highest and lowest log_2_FC values between GB PN and LGG, including functions and pathways (Table S5); over-representation analysis (ORA) using gene ontology (GO) enrichment and Kyoto Encyclopedia of Genes and Genomes (KEGG) pathway analysis showing the pathways and functions with the highest numbers of over- and underexpressed proteins in the four HGG subgroups (compared to LGG) (Table S6); differentially expressed proteins between GB CL and LGG (Table S7); differentially expressed proteins with the highest and lowest log_2_FC values between GB CL and LGG, including functions and pathways (Table S8); differentially expressed proteins between GB MES and LGG (Table S9); differentially expressed proteins with the highest and lowest log_2_FC values between GB MES and LGG, including functions and pathways (Table S10); differentially expressed protein between IDH HGG and LGG (Table S11); differentially expressed protein between IDH HGG and LGG, including functions and pathways (Table S12); comparison of DEPs between individual HGG subgroups (Table S13); comparison of DEPs between each HGG subgroup and the three other pooled HGG subgroups (Table S14); univariable Cox regression analysis of differentially expressed proteins and survival (Table S15) (XLSX)

## Abbreviations and glossary

Å: Ångstrom
AC-like: astrocyte-like
AKT: protein kinase B
ALT: alternative lengthening of telomere
ATP: adenosine triphosphate
*ATRX*: alpha-thalassemia/mental retardation X-linked
*CDKN2A*: *cyclin-dependent kinase inhibitor 2A*
CNS: central nervous system
CNV: copy number variations
DEP: differentially expressed protein
DKFZ: Deutsches Krebsforschungszentrum
EKNZ: Ethikkommission Nordwest- und Zentralschweiz
EMT: epithelial-to-mesenchymal transition
GB: glioblastoma
GB CL: glioblastoma, *IDH*-wildtype, subtype RTK II (classical)
GB MES: glioblastoma, *IDH*-wildtype, subtype mesenchymal GB PN glioblastoma, *IDH*-wildtype, subtype RTK I (proneural)
GO: gene ontology
GSEA: gene set enrichment analysis
HGG: high-grade glioma
HPLC: high-performance liquid chromatography
*IDH 1/2*: *isocitrate dehydrogenase 1/2*
*IDH* CODEL: oligodendroglioma, IDH mutant and 1p/19q-codeleted
*IDH* HGG: high-grade astrocytoma, *IDH* mutant
*IDH* LGG: astrocytoma, *IDH* mutant
KEGG: Kyoto Encyclopedia of Genes And Genomes
LC: liquid chromatography
LGG: low-grade glioma
log_2_FC: log_2_ fold change
M: molarity
MALDI-MSI: matrix-assisted laser desorption/ionization mass spectrometry imaging
MES-like: mesenchymal-like
MS: mass spectrometry
mTOR: mammalian target of rapamycin
NF1: neurofibromin 1
NPC-like: neural progenitor-like
OPC-like: oligodendrocyte-progenitor-like
ORA: over-representation analysis
PCA: principal component analysis
PI3K: phosphatidylinositol-3 kinase
PMT: proneural-to-mesenchymal transition
PDGFR: platelet-derived growth factor receptor
PPI: protein–protein interaction
PTEN: phosphatase and TENsin homologue
RAS: rat sarcoma
RTK I and II: receptor-tyrosine kinase I and I
TCGA: The cancer genome atlas
TERT: telomerase reverse transcriptase
TFA: trifluoroacetic acid
TMT: tandem mass tag
TP53: tumor protein 53
UMAP: uniform manifold approximation and projection
WHO: World Health Organization

## Proteins and short names

AIG1: androgen-induced gene 1 protein
NES: nestin
ANXA5: annexin A5
PTPRC: receptor-type tyrosine-protein phosphatase C (CD45)
CENPV: centromere protein V
RBP1: RalA-binding protein 1
COG8: conserved oligomeric Golgi complex subunit 8
REN3A: regulator of nonsense transcripts 3A
DDX3Y: ATP-dependent RNA helicase DDX3Y
S10AD: protein S100-A13
EGFR: epidermal growth factor receptor
SE6L1: seizure 6-like protein
GBG4: guanine nucleotide-binding protein G­(I)/G­(S)/G­(O) subunit gamma-4
SEPT3: neuronal-specific septin-3
GPNMB: glycoprotein NMB
SYVM: valine-tRNA ligase, mitochondrial
H13: histone H1.3
TAGL2: transgelin-2
H15: histone H1.5
TENA: tenascin C
HG2A: HLA class II histocompatibility antigen gamma chain
TM245: transmembrane protein 245
HSPB6: heat shock protein beta-6
TYB10: thymosin beta 10 (TMSB10)
IQGA1: Ras GTPase-activating-like protein IQGAP1
TRIM24: transcription intermediary factor 1-alpha
LEG3: galectin-3 (LGALS3)
TXTP: tricarboxylate transport protein, mitochondrial
MET15: 12S rRNA N4-methylcytidine (m4C) methyltransferase
VIM: vimentin
MOES: moesin
ZDH14: palmitoyltransferase ZDHHC14
