# Supplementary material for: Quantitative Proteomic Analysis Reveals Different Functional Subtypes among IDH-Wildtype Glioblastoma
Source: J Proteome Res. 2025 Jun 16;24(7):3610–24. doi: 10.1021/acs.jproteome.5c00199 (PMC12235693; doi:10.1021/acs.jproteome.5c00199)
Supplement: Supplementary file 1 [file pr5c00199_si_002.pdf]

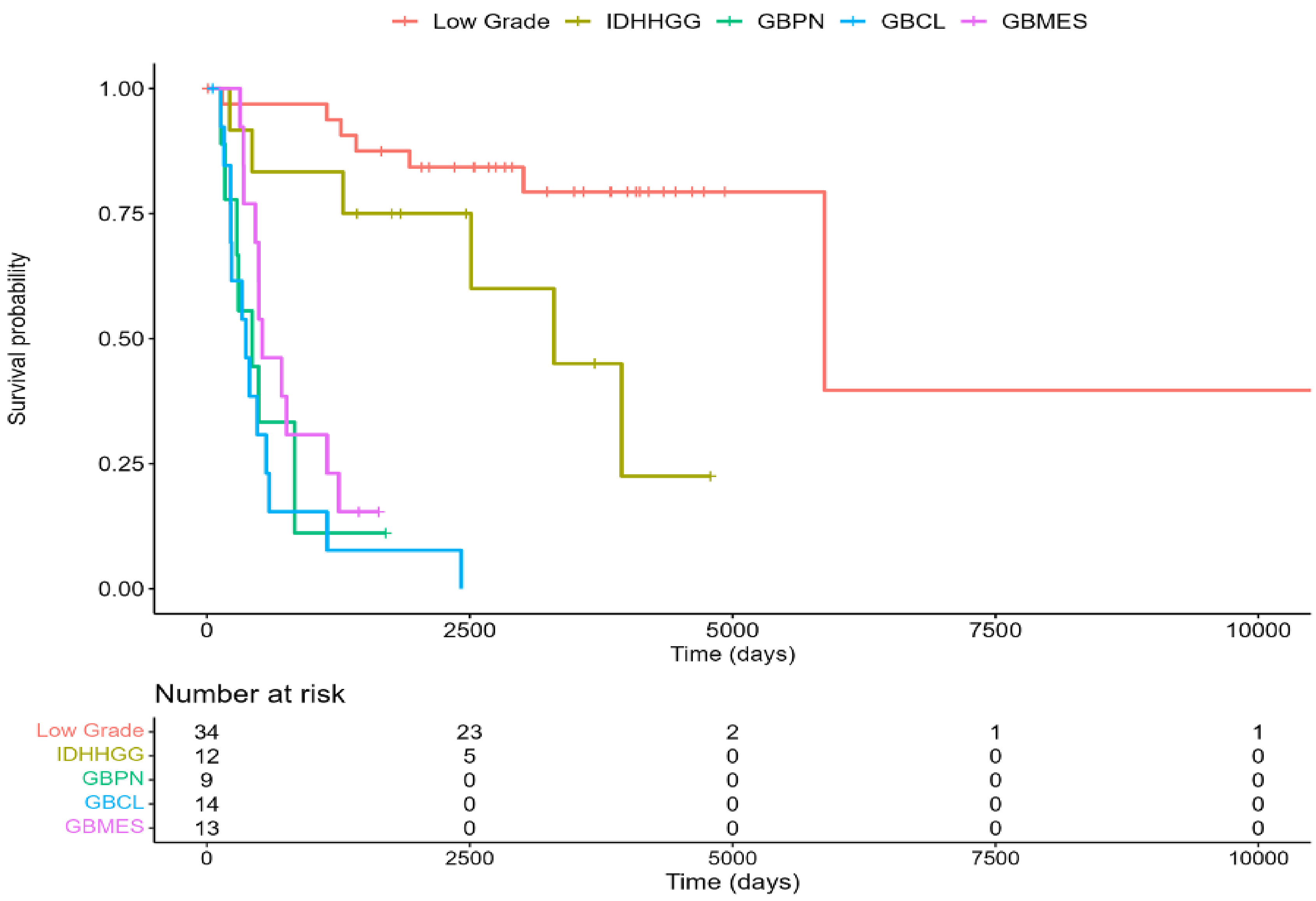

Supporting Figure S1 : Kaplan-Meier curve showing the overall survival of the 6 glioma subgroups (Note: the two LGG subgroups are given as one pooled group (Low grade)).

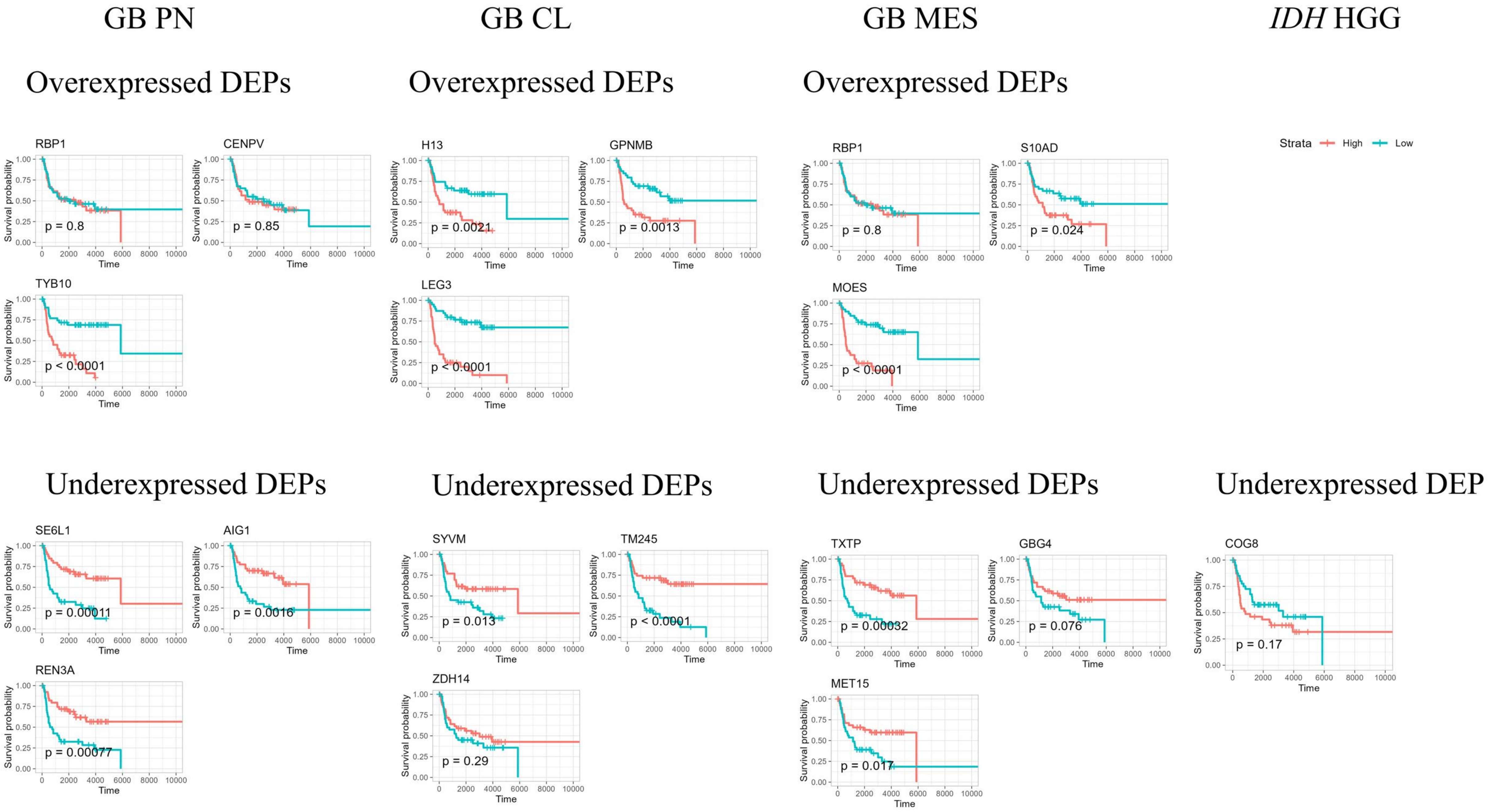

Supporting Figure S2: Association of the three most overexpressed and three most underexpressed DEPs from each subgroup with overall survival. (DEP abundance was split into high and low expression groups at the median, Kaplan-Meier survival analysis was done to estimate survival curves, and the log-rank test was used to assess differences between the groups).

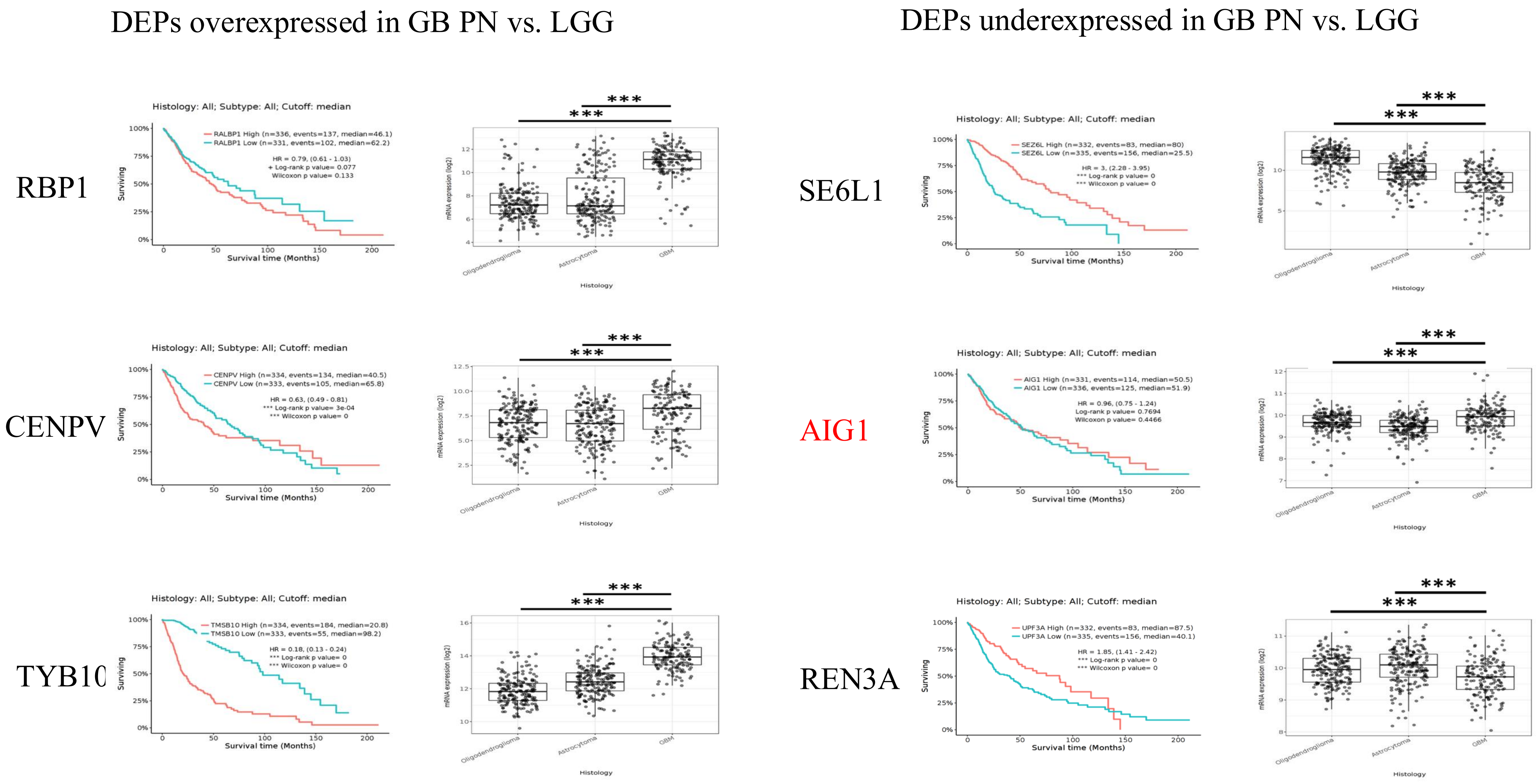

Supporting Figure S3: TCGA data using the TCGA GBM-LGG cohort showing the mRNA expression levels of the DEPs most over- and underexpressed in GB PN, and association of high and low mRNA levels of these DEPs with survival. Note: in red listed are the proteins that have contradicting expression levels and association withsurvival in the TCGA GBM-LGG database compared to our data (www.tcga.gov).

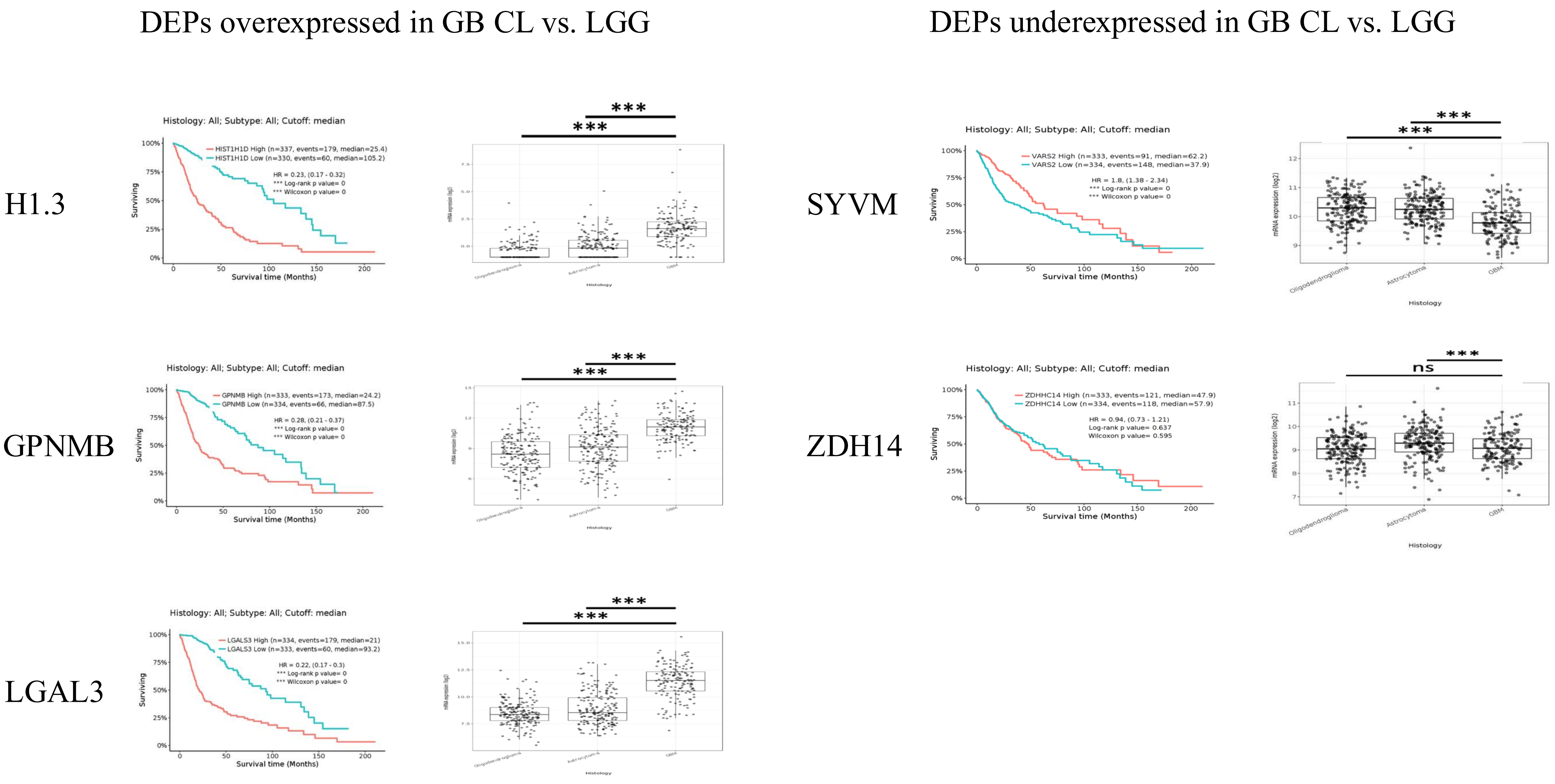

Supporting Figure S4: TCGA data using the TCGA GBM-LGG cohort showing the mRNA expression levels of the DEPs most over- and underexpressed in GB CL, and association of high and low mRNA levels of these DEPs with survival. There were no results available for mRNA expression of TM245 in glioma (www.tcga.gov).

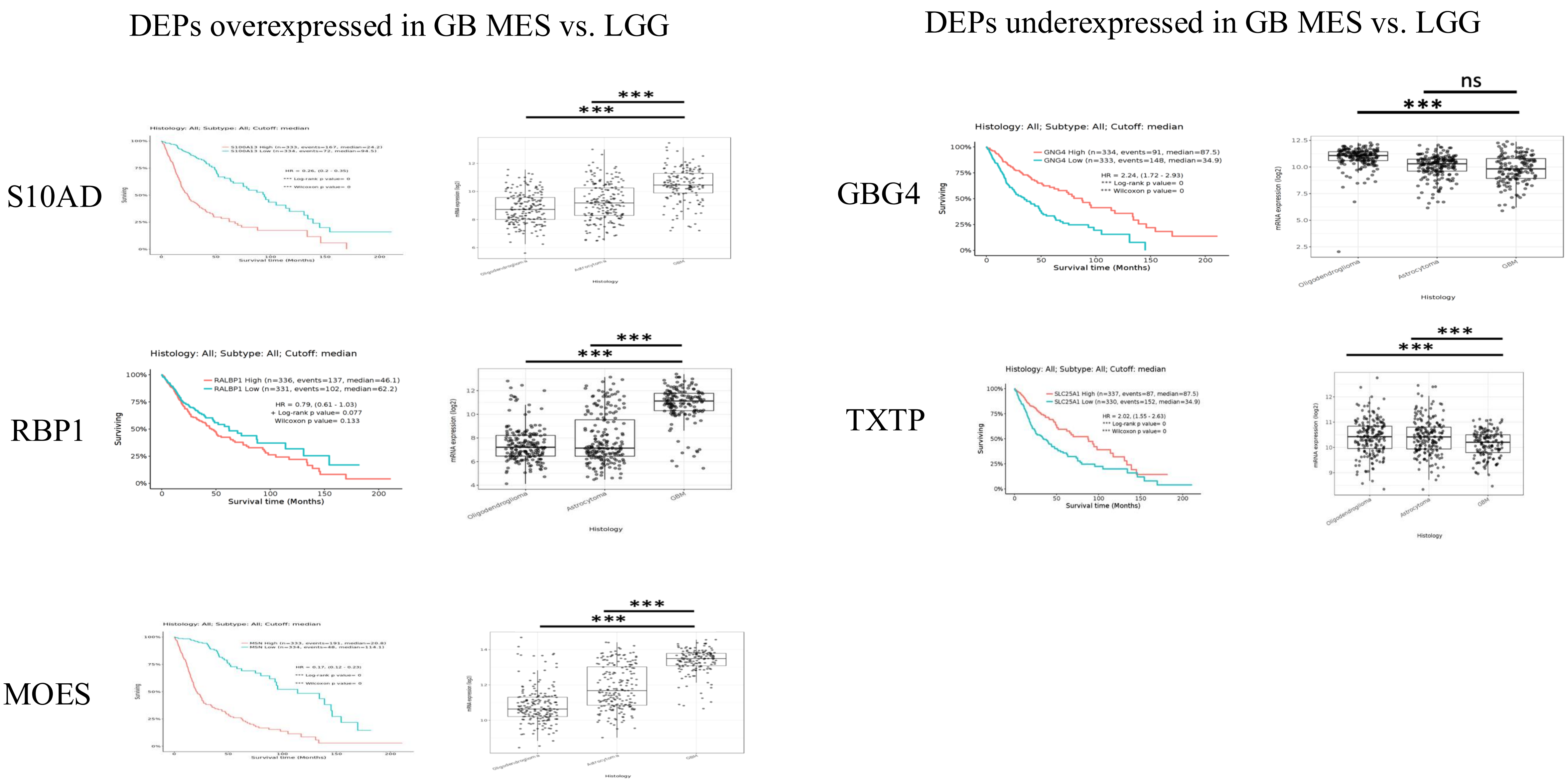

Supporting Figure S5: TCGA data using the TCGA GBM-LGG cohort showing the mRNA expression levels of the DEPs most over- and underexpressed in GB MES compared to LGG, and association of high and low mRNA levels of these DEPs with survival. There were no results available for mRNA expression of MET15 in glioma (www.tcga.gov).

DEP underexpressed in *IDH* HGG vs. LGG

COG8

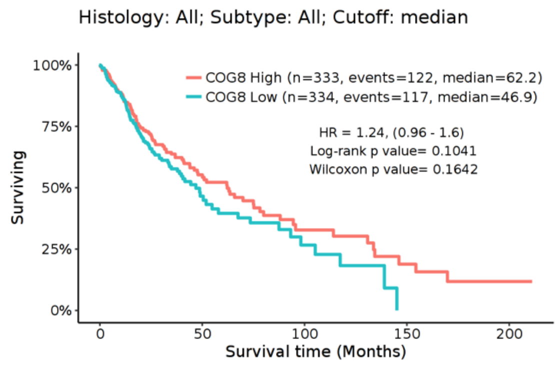

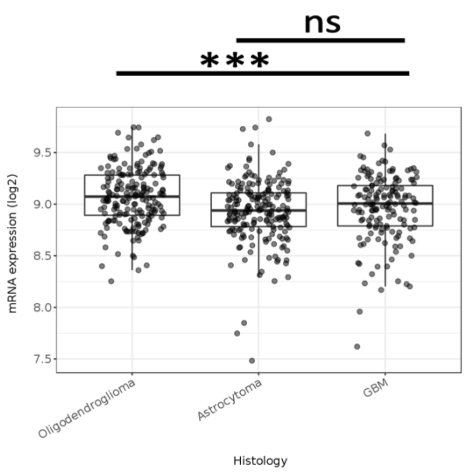

Supporting Figure S6: TCGA data using the TCGA GBM-LGG cohort showing the mRNA expression level of the only DEP significantly underexpressed in IDH HGG compared to LGG, and association of high and low mRNA levels of this DEP with survival (www.tcga.gov).

Inhibitor response assay with GB1107 -> targeting LEG3 – overexpressed in GB CL

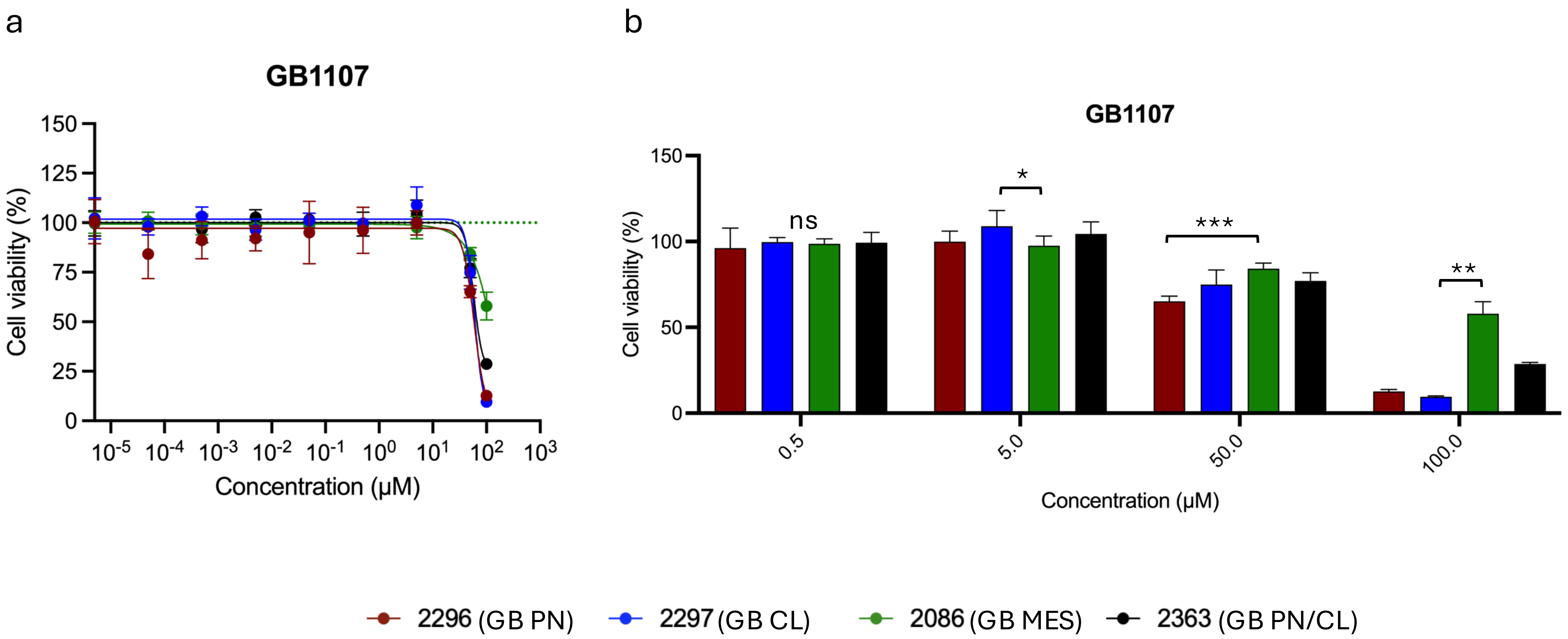

Supporting Figure S7: Inhibitor response assay using GB1107 targeting LEG3

a) Dose response curves for dissociated tumor cells of patients representative of GB PN (red), GB CL (blue), GB MES (green) and GB PN/CL (black).

b) Percentage of cell viability for the four highest concentrations of GB1107 applied in inhibitor assays. Results at 500 µM not shown due to precipitation. * $p < 0.05$, ** $p < 0.01$, *** $p < 0.001$

Inhibitor response assay with Glembatumumab vedotin -> targeting GPNMB – overexpressed in GB CL

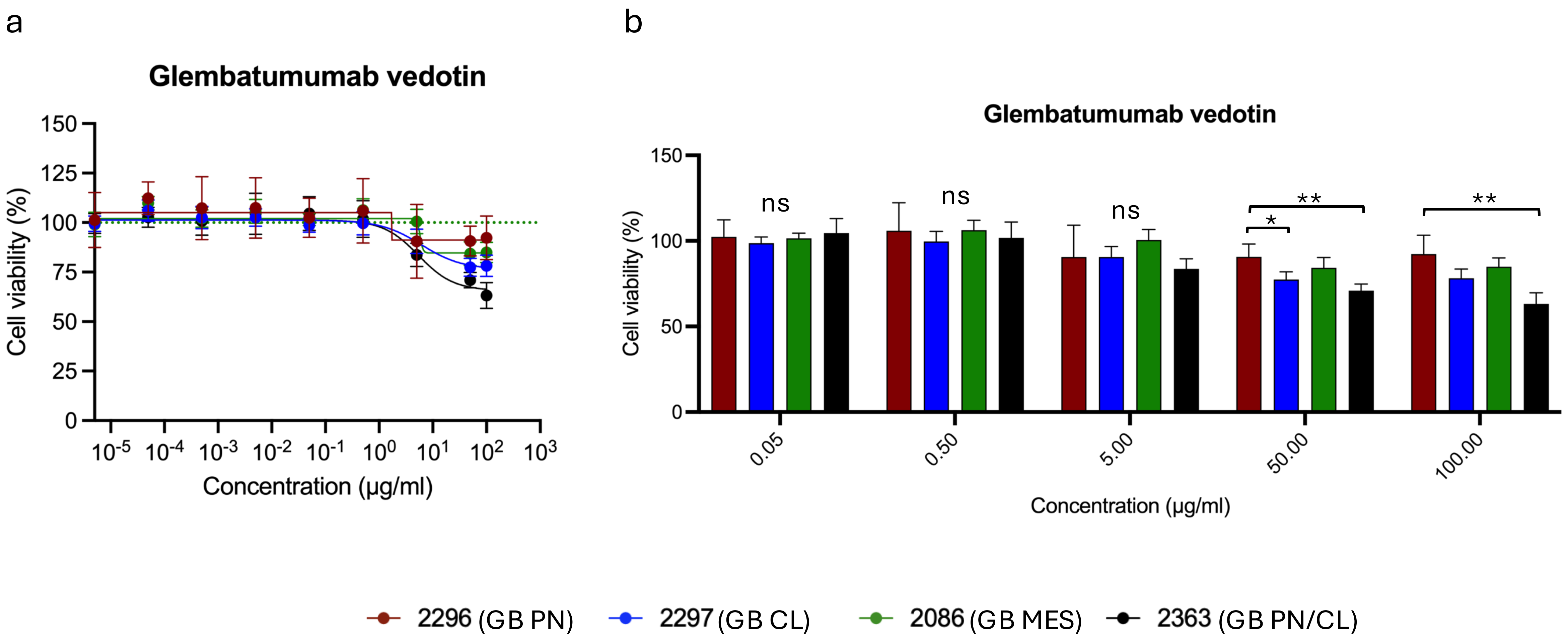

Supporting Figure S8: Inhibitor response assay using Glembatumumab vedotin targeting GPNMB

a) Dose response curves for dissociated tumor cells of patients representative of GB PN (red), GB CL (blue), GB MES (green) and GB PN/CL (black).

b) Percentage of cell viability for the five highest concentrations of Glembatumumab vedotin applied in inhibitor assays. * $p < 0.05$, ** $p < 0.01$, *** $p < 0.001$

Inhibitor response assay with Vorinostat -> targeting H13 – overexpressed in GB CL

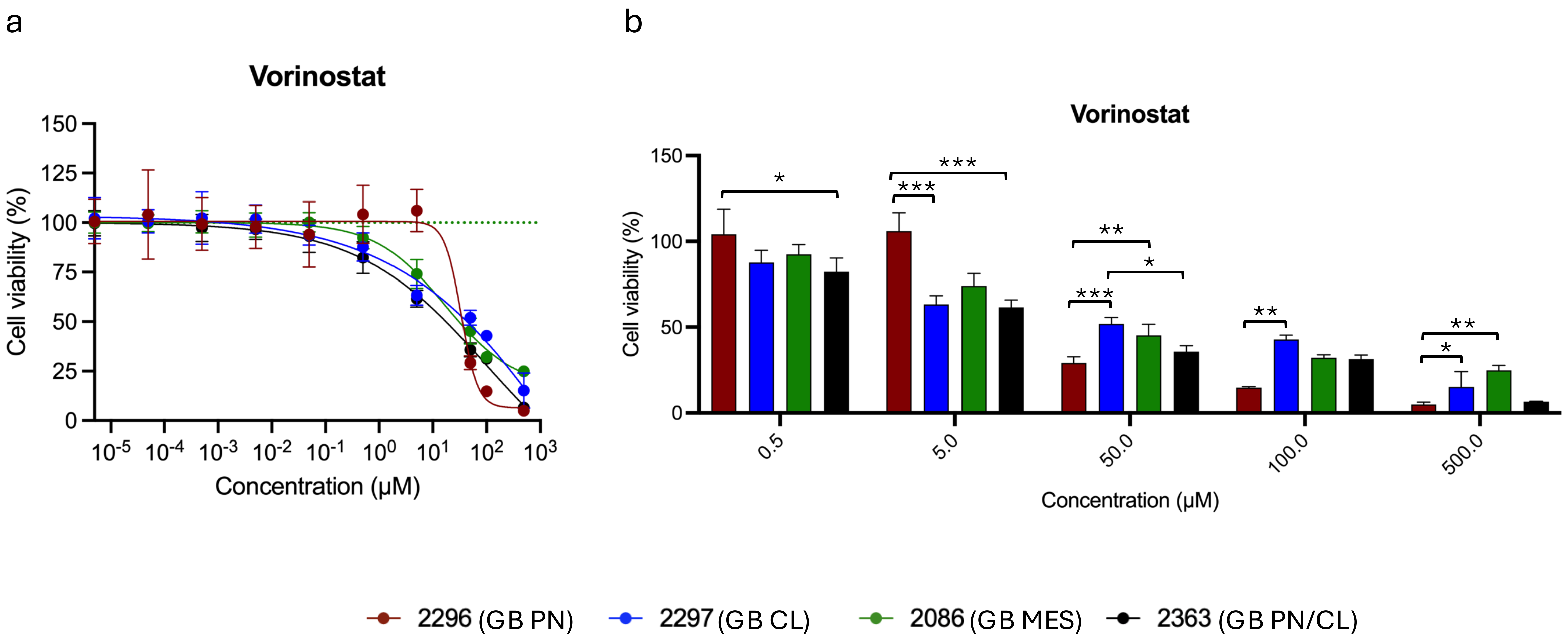

Supporting Figure S9: Inhibitor response assay using Vorinostat targeting H13

a) Dose response curves for dissociated tumor cells of patients representative of GB PN (red), GB CL (blue), GB MES (green) and GB PN/CL (black).

b) Percentage of cell viability for the five highest concentrations of Vorinostat applied in inhibitor assays. * $p < 0.05$, ** $p < 0.01$, *** $p < 0.001$

Inhibitor response assay with GSK-923295 -> targeting CENPV – overexpressed in GB PN

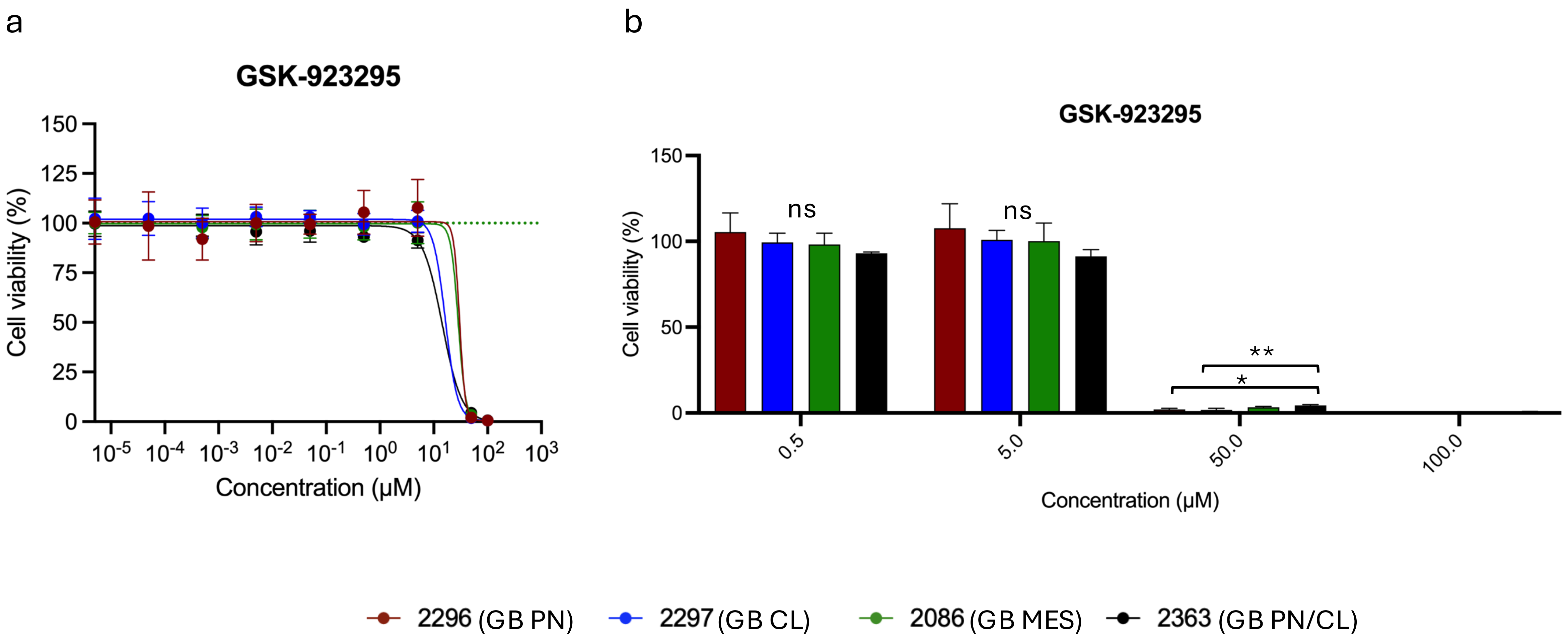

Supporting Figure S10: Inhibitor response assay using GSK-923295 targeting CENPV
a) Dose response curves for dissociated tumor cells of patients representative of GB PN (red), GB CL (blue), GB MES (green) and GB PN/CL (black).
b) Percentage of cell viability for the four highest concentrations of GSK-923295 applied in inhibitor assays. Results at 500 µM not shown due to precipitation. * $p < 0.05$, ** $p < 0.01$, *** $p < 0.001$

Inhibitor response assay with Amlexanox -> targeting S10AD – overexpressed in GB MES

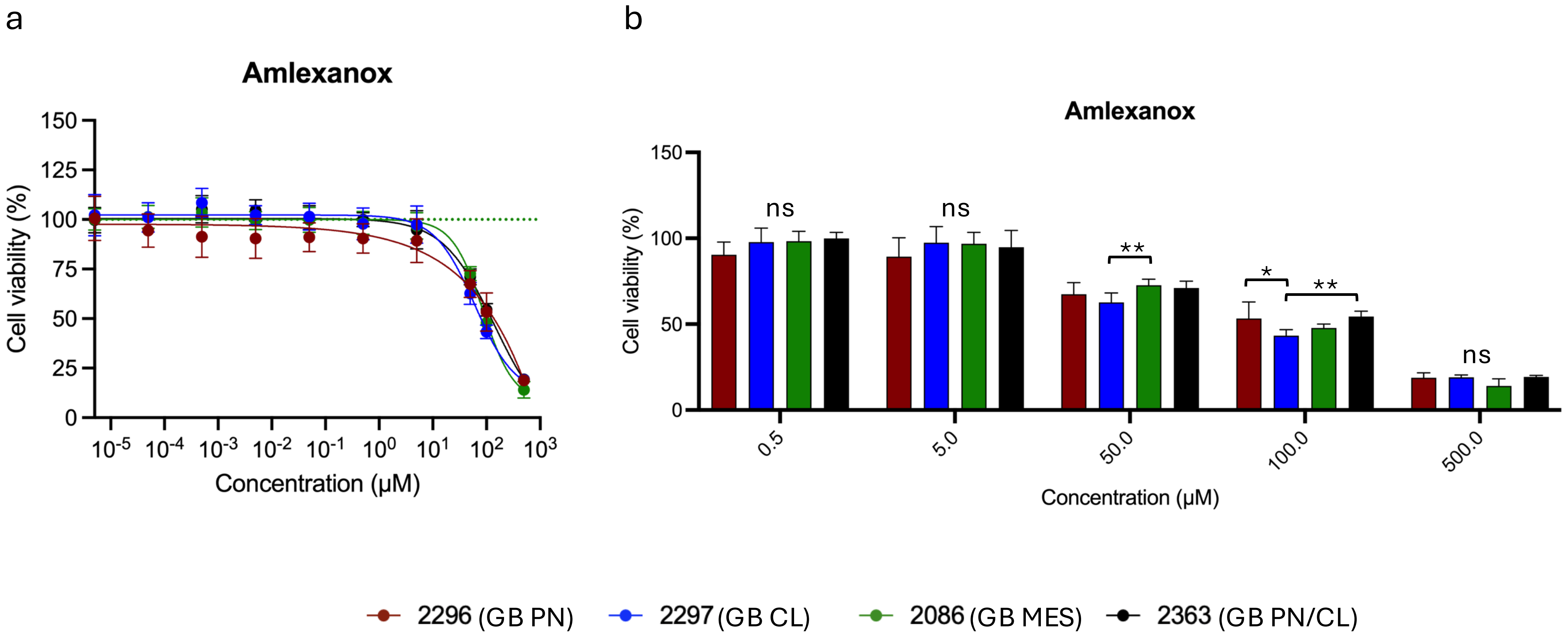

Supporting Figure S11: Inhibitor response assay using Amlexanox targeting S10AD

a) Dose response curves for dissociated tumor cells of patients representative of GB PN (red), GB CL (blue), GB MES (green) and GB PN/CL (black).

b) Percentage of cell viability for the five highest concentrations of Amlexanox applied in inhibitor assays. * $p < 0.05$, ** $p < 0.01$, *** $p < 0.001$

Inhibitor response assay with NSC668394 -> targeting MOES – overexpressed in GB MES

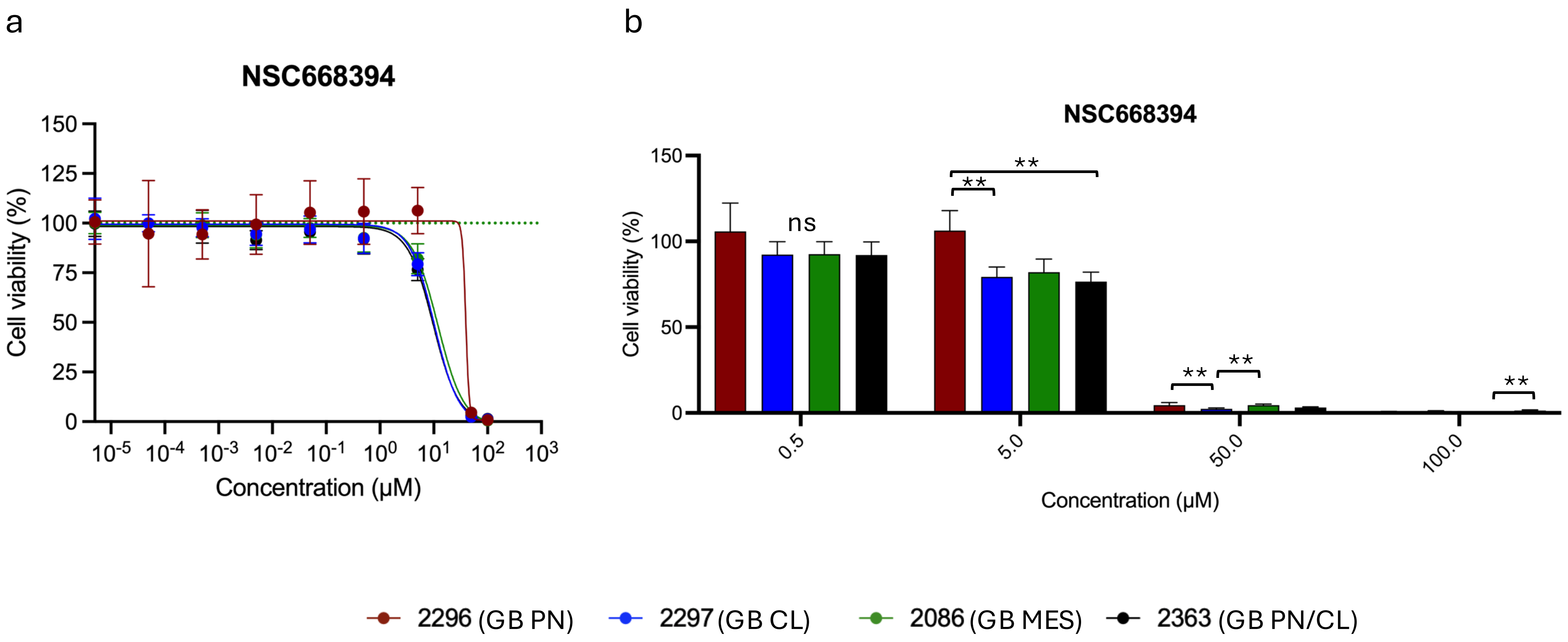

Supporting Figure S12: Inhibitor response assay using NSC668394 targeting MOES

a) Dose response curves for dissociated tumor cells of patients representative of GB PN (red), GB CL (blue), GB MES (green) and GB PN/CL (black).

b) Percentage of cell viability for the four highest concentrations of NSC668394 applied in inhibitor assays. Results at 500 µM not shown due to precipitation. * $p < 0.05$, ** $p < 0.01$, *** $p < 0.001$

Inhibitor response assay with Fasudil HCl -> targeting MOES – overexpressed in GB MES

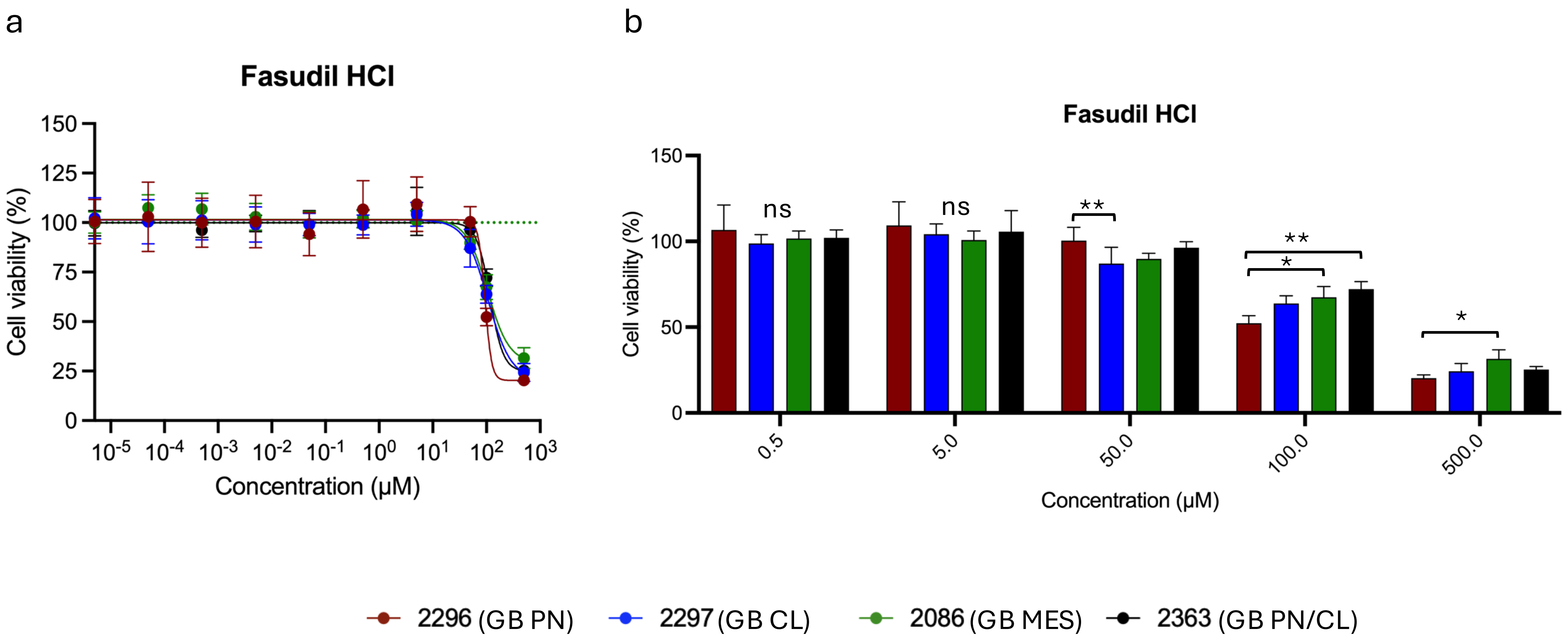

Supporting Figure S13: Inhibitor response assay using Fasudil HCl targeting MOES

a) Dose response curves for dissociated tumor cells of patients representative of GB PN (red), GB CL (blue), GB MES (green) and GB PN/CL (black).

b) Percentage of cell viability for the five highest concentrations of Fasudil HCl applied in inhibitor assays. * $p < 0.05$, ** $p < 0.01$, *** $p < 0.001$
